# Development and validation of a multi-lingual online questionnaire for surveying the COVID-19 prevention and control measures used in global workplaces

**DOI:** 10.1186/s12889-022-12500-w

**Published:** 2022-01-12

**Authors:** Carolyn Ingram, Yanbing Chen, Conor Buggy, Vicky Downey, Mary Archibald, Natalia Rachwal, Mark Roe, Anne Drummond, Carla Perrotta

**Affiliations:** 1 School of Public Health, Physiotherapy, and Sports Science, University College Dublin, D04 V1W8 Dublin, Ireland; 2 Centre for Safety & Health at Work, School of Public Health, Physiotherapy, and Sports Science, University College Dublin, D04 V1W8 Dublin, Ireland

**Keywords:** Occupational safety and health, COVID-19, Psychometric validation, Online survey, Infection prevention and control

## Abstract

**Background:**

Despite widespread COVID-19 vaccination programs, there is an ongoing need for targeted disease prevention and control efforts in high-risk occupational settings. This study aimed to develop, pilot, and validate an instrument for surveying occupational COVID-19 infection prevention and control (IPC) measures available to workers in diverse geographic and occupational settings.

**Methods:**

A 44-item online survey was developed in English and validated for face and content validity according to literature review, expert consultation, and pre-testing. The survey was translated and piloted with 890 workers from diverse industries in Canada, Ireland, Argentina, Poland, Nigeria, China, the US, and the UK. Odds ratios generated from univariable, and multivariable logistic regression assessed differences in ‘feeling protected at work’ according to gender, age, occupation, country of residence, professional role, and vaccination status. Exploratory factor analysis (EFA) was conducted, and internal consistency reliability verified with Cronbach’s alpha. Hypothesis testing using two-sample t-tests verified construct validity (i.e., discriminant validity, known-groups technique), and criterion validity.

**Results:**

After adjustment for occupational sector, characteristics associated with feeling protected at work included being male (AOR = 1.88; 95% CI = 1.18,2.99), being over 55 (AOR = 2.17; 95% CI = 1.25,3.77) and working in a managerial position (AOR = 3.1; 95% CI = 1.99,4.83). EFA revealed nine key IPC domains relating to: environmental adjustments, testing and surveillance, education, costs incurred, restricted movements, physical distancing, masking, isolation strategies, and areas for improvement. Each domain showed sufficient internal consistency reliability (Cronbach’s alpha ≥0.60). Hypothesis testing revealed differences in survey responses by country and occupational sector, confirming construct validity (*p* < 0.001), criterion validity (*p* = 0.04), and discriminant validity (*p* < 0.001).

**Conclusions:**

The online survey, developed in English to identify the COVID-19 protective measures used in diverse workplace settings, showed strong face validity, content validity, internal consistency, criterion validity, and construct validity. Translations in Chinese, Spanish, French, Polish, and Hindi demonstrated adaptability of the survey for use in international working environments. The multi-lingual tool can be used by decision makers in the distribution of IPC resources, and to guide occupational safety and health (OSH) recommendations for preventing COVID-19 and future infectious disease outbreaks.

**Supplementary Information:**

The online version contains supplementary material available at 10.1186/s12889-022-12500-w.

## Background

Relatively few infectious individuals (~ 10%) are responsible for most (~ 80%) of local COVID-19 transmission [1]. Environmental factors likely contribute to COVID-19 clusters, or ‘superspreading’ events. Crowded indoor settings, poor ventilation, long contact exposures, and/or frequent interactions with the general public create high or medium-risk working environments for COVID-19 [2, 3]. Workplaces such as hospitals, care homes, schools, building sites, food-processing plants, and high-volume retail outlets possess such environmental features that facilitate explosive COVID-19 clusters [4]. Comprehensive occupational COVID-19 infection prevention and control (IPC) measures can prevent these events, ensuring the protection of workers, their families, and surrounding communities. Effective measures include swift and thorough contact tracing and case isolation, effective personal protective equipment (PPE), testing, worker bubbles, and improved ventilation and air quality [5–8]. Relying solely on a single control measure like masking is not enough to prevent COVID-19 clusters in high-risk workplace environments [6, 7].

Many countries have been active in creating COVID-19 occupational safety and health (OSH) policies and guidance involving these measures [9]. Nevertheless, data indicate that workers remain at disproportionate risk to COVID-19 in settings where vaccination rates lag and cases surge [10–12]. The United States’ Occupational Safety and Health Administration (OSHA) issued its first mandatory workplace safety rules aimed at protecting healthcare workers from COVID-19 in June 2021 [13]. Yet these rules do not protect workers in other essential industries who face rising case numbers, stalled vaccinations [14], loosened mask requirements [15], and push back against occupational COVID-19 safety regulations from the national business community [16]. In Europe, as businesses reopen and travel restrictions ease [17], countries continue to experience workplace outbreaks [10, 11]. Case numbers amongst oil sands workers in Canada grew so high in May 2021 that legislators had to declare a state of local emergency [18]; and in India, an unprecedented surge in cases March–April 2021 strained IT firms who struggled to protect the health of employees while maintaining business continuity [19]. These events highlight the ongoing need for disease prevention and control guidance and resources in high-risk occupational settings, particularly as workers have expressed anxiety and concern about becoming infected with COVID-19 and endangering co-workers, customers, and family [20].

Occupational hygienists have called for the production of appropriate tools to effectively transfer safe IPC knowledge and practice to employers and workers [21]. At the international-level, the International Labour Organization (ILO) provides a database with updates on rapidly changing occupational policy responses to the COVID-19 crisis [22]. At the individual-level, to our knowledge, no validated tool exists for surveying workers’ awareness of and satisfaction with the implementation of these policies. As research reveals gaps in policymakers’ and workers’ perceptions of acceptable COVID-19 safety at work, it is important to constructively engage with workers in order to fast track acceptable and feasible OSH solutions [23]. Understanding protective measures in place can, by helping to untangle potential causes of outbreaks, aid governments trying to contain or prevent COVID-19 surges around the world. Identifying gaps in workplace safety response can help to direct the distribution of disease prevention and control resources and raise awareness of effective COVID-19 safety measures.

This study aimed to develop, pilot, and validate an instrument for identifying occupational COVID-19 IPC measures available to workers from diverse geographic and occupational settings. By allowing researchers to measure workers’ perceptions of the level of COVID-19 protections in place, the survey instrument will have important implications for worker protection from COVID-19 and future infectious disease outbreaks.

## Methods

**Fig. 1:**
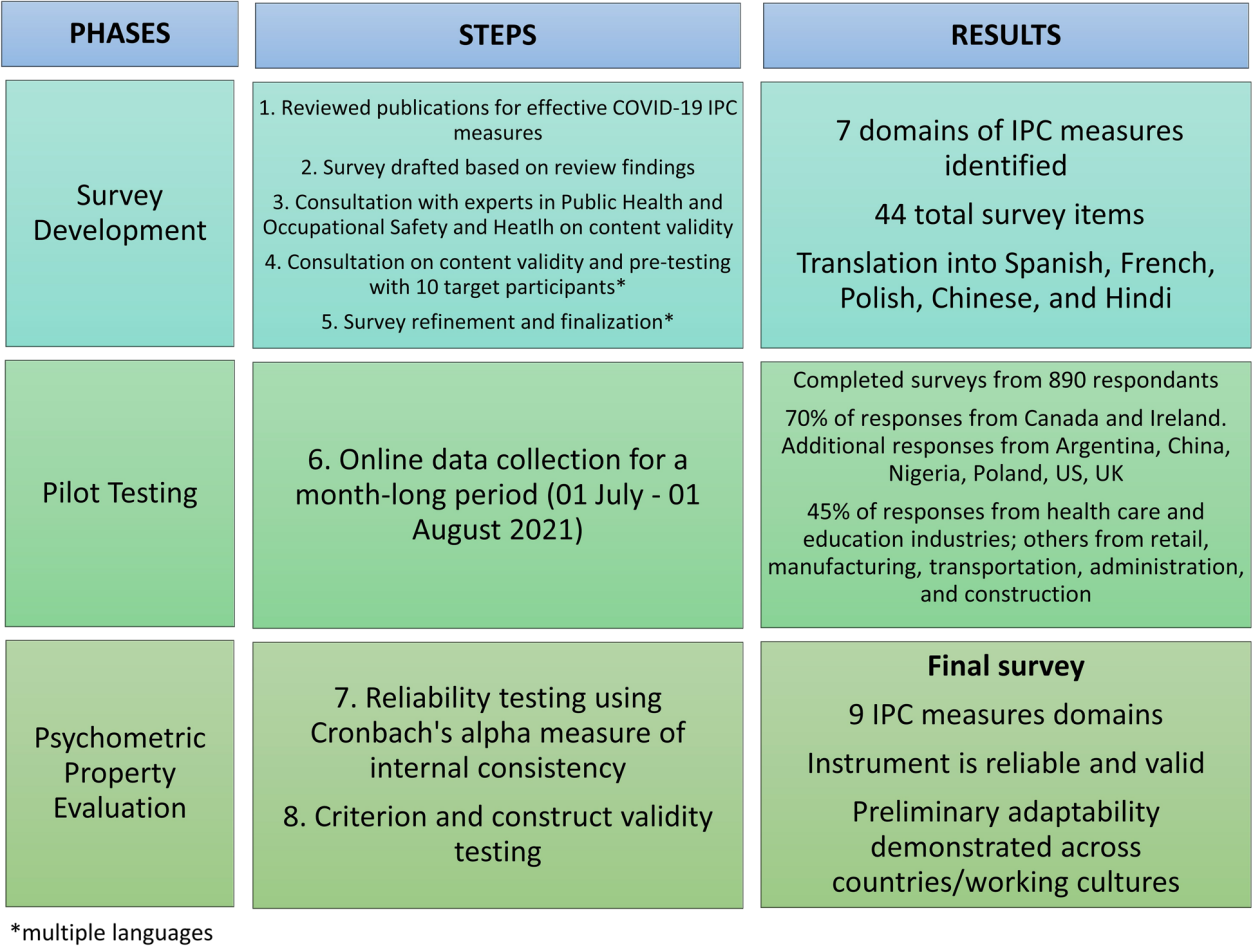
Methodological steps taken to design, pilot, and validate a multi-lingual online survey for identifying occupational COVID-19 IPC measures used in international workplace settings. *multiple languages

**Fig. 2:**
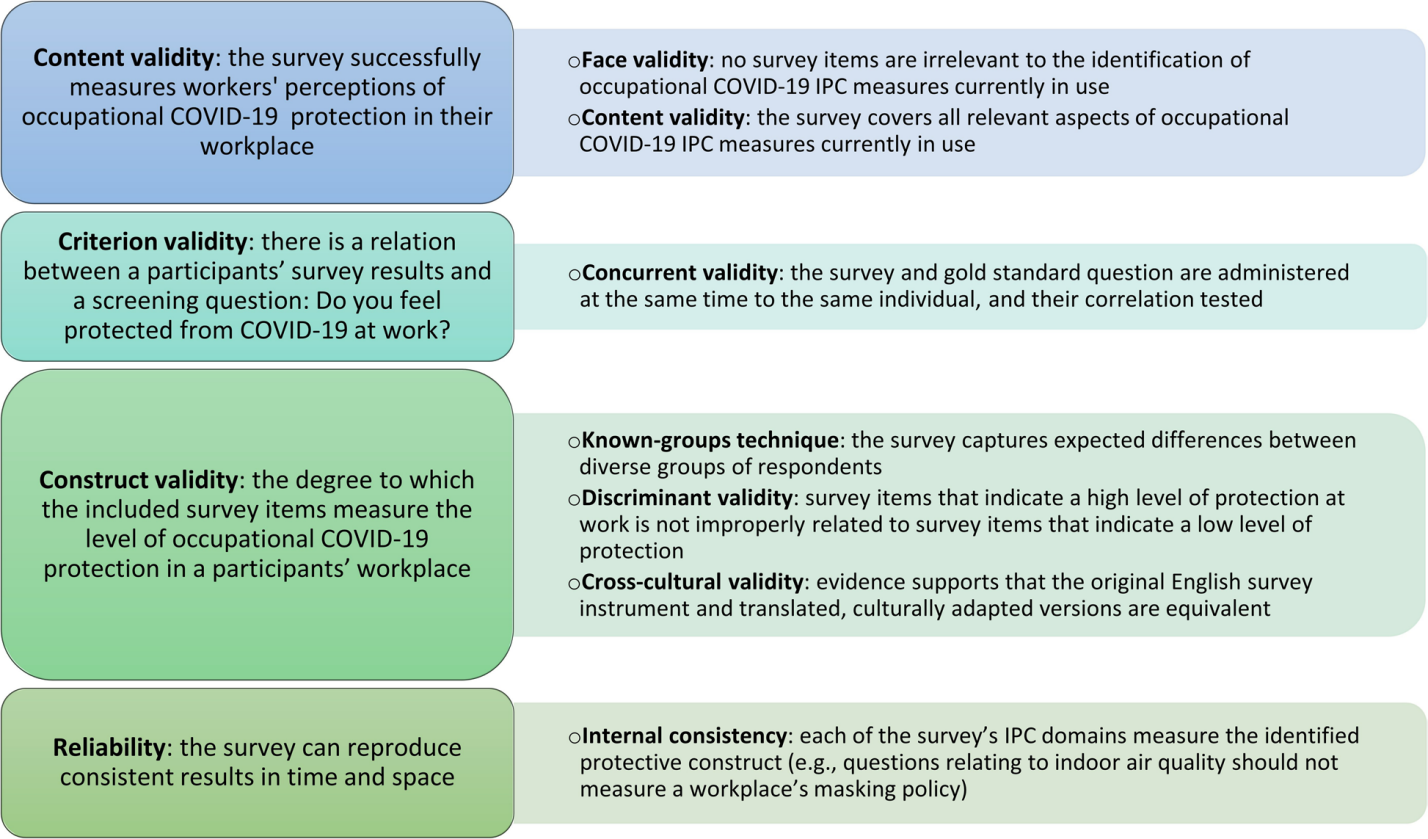
Validity and reliability measurement of a multi-lingual online survey for identifying occupational COVID-19 IPC measures used in international workplace settings

This cross-sectional, pilot study was conducted in three phases: 1) survey development, 2) pilot testing, and 3) psychometric property evaluation. Specific methodological steps taken during each phase are outlined in Fig. 1. Psychometric properties assessed as part of the study are defined in Fig. 2.

### Phase 1. Survey development

#### Creating the survey

From February to April 2019, the researchers conducted an extensive review of relevant research literature and meta-analysis to investigate the effectiveness of measures to prevent and control COVID-19 outbreaks in global workplace settings. Results from meta-analyses showed that combined IPC measures resulted in lower post-intervention employee COVID-19 positivity rates (0.2% positivity; 95% CI 0,0.4%) than single measures such as asymptomatic PCR testing (1.7%; 95% CI 0.9,2.9%) and universal masking (24%; 95% CI 3.4,55.5%). Specifically, combinations of (1) basic preventive measures (i.e., masking, hand hygiene, and social distancing), (2) surveillance measures, (3) outbreak investigations and response, (4) environmental adjustments, and (5) education initiatives were shown to effectively prevent workplace outbreaks [6]. These categories became the framework for the development of a survey allowing researchers to measure the level of occupational COVID-19 protection available to workers from diverse geographic and occupational settings.

A preliminary questionnaire in English was designed on Qualtrics^XM^ (Provo, UT) online survey platform with questions pertaining to:

1. Basic Demographics (age, gender, country of residence, education);
2. Employment Demographics (workplace category, industry, size, current role);
3. Basic preventive measures (hand hygiene, barriers, social distancing, masking, etc.);
4. Environmental adjustments (temperature, ventilation, air quality monitoring);
5. Surveillance (type of testing, access to testing, self-isolation requirements, syndromic surveillance);
6. Contact tracing;
7. PPE;
8. Education and training; and
9. Vaccination.

Questions were designed for actively working employees, managers, and occupational safety and health professionals from any country. Skip patterns were enabled based on participant characteristics. For example, managers and employees were given access to separate question tracks, and only participants that selected “Healthcare and Social Assistance” as their occupational sector could access questions on medical PPE. As the survey was intended for internationally and occupationally diverse participants, survey questions were worded as generally and simply as possible.

#### Establishing content validity

The preliminary survey was independently reviewed by five multinational experts in Public Health and Occupational Safety and Health to ensure face validity (that the questions adequately met the study’s aims) and content validity (to ensure that questions were all relevant to the study’s aims). Once independent review was complete, experts convened for two, one-hour meetings with research team members to discuss findings. Survey items that did not reach consensus during the first meeting were removed, modified, or reworded by the researchers and presented again for expert review. During the second meeting, experts reached consensus on all survey items as well as on general format, language, and response options.

Prior to dissemination, the Qualtrics survey was pre-tested with 10 actively working individuals from Ireland and the United States. Researchers met one-one-one with construction (*n* = 1), retail (*n* = 2), education (*n* = 3), manufacturing (n = 1), and healthcare (n = 3) employees to ensure adequate comprehension of the information sheet, survey items, and response options. Pre-testing data was reviewed, and the survey adapted based on participant feedback. Researchers added questions pertaining to a pandemic transition phase (i.e., measures that workers would like to see upheld/removed as vaccinations increase), and whether or not workers feel adequately protected from COVID-19 at work. Pre-testing allowed researchers to identify and correct errors in skip patterns.

#### Translating the survey

The survey instrument, once finalized in English, was translated into six languages, and adapted for working cultures across countries based on input from key informants. This process followed standard techniques so far as the project scope allowed [24]:

1. **Forward translation** of the survey into Spanish (Latin American and Argentinian dialects), French, Polish, Hindi, and Chinese was conducted by members of the research team with native-level fluency in these languages.
2. **Independent reviewing** of translated versions by a native-level speaker (generally an acquaintance of the original translator living in a target country) ensured semantic and conceptual relevance.
3. **Back-translation** followed a two-stage model of cost-effective translation recommended by Taylor et al. [25] involving (1) machine translation and (2) proof reading and editing by a human translator. Research suggests that 90% of machine translation using Google Translate is acceptable in terms of quality and safety [26]. Thus, back-translation of each survey was conducted using Google Translate™, after which a native researcher identified and edited potential discrepancies between the original and translated survey.
4. **Pre-testing** of each translation with at least one actively working native speaker from outside of the public/occupational health sphere allowed researchers to assess ease of comprehension of translated items. Participants commented on items they found unclear or irrelevant, generating further improvements until translations were deemed adequately culturally adapted for pilot testing.

### Phase 2. Pilot testing

#### Subjects, sampling, and recruitment

To pilot the survey, individuals who were actively working in July 2021 in Ireland, Canada, the US, the UK, Argentina, China, India, Nigeria, or Poland were targeted. These countries were selected (1) to represent a diverse array of pandemic stages and vaccination rates, (2) to facilitate collaboration with professional contacts of the research team, and (3) based on national language. Included participants were working individuals aged 18 or older with literacy in English, French, Spanish, Hindi, Polish, or Chinese. Participants who were currently working full-time from home were also included. However, they were directed to a shortened version of the questionnaire with questions pertaining to (1) basic demographics, (2) employment demographics, and (3) vaccination status. Because working from home is a safety measure in itself, the inclusion of said participants was considered relevant to the survey’s objectives. Individuals who were not currently working (i.e., unemployed, on leave, retired) were excluded from the study.

Target participants were recruited between 01 July and 01 August 2021 through non-probability convenience sampling techniques. To ensure a geographically and occupationally diverse sample, researchers advertised the survey link through multiple recruitment channels in a manner similar to McRobert et al. [27]:

- **Formal recruitment channels:** OSH organizations and national trade unions of target countries were contacted by the researchers and, if willing to collaborate, sent the survey link to all constituents via email;
- **Social media groups:** Researchers joined relevant occupational LinkedIn and Facebook groups and posted the survey link into those groups; and
- **Personal social media:** Researchers’ posted the link to their own social media profiles (Facebook, LinkedIn, Twitter, WhatsApp).

The multi-modal online participant recruitment strategy was approved by the University College Dublin Human Research Ethics Committee (LS-E-21-138-Perrotta) and followed recommended procedures for online surveys. By keeping careful records of where, when, and by whom participants were contacted, and the number of participants contacted [28], partnering with other organizations and encouraging peer-led snowball sampling [29], and using an open access, ethically approved, and user optimized instrument [30], the researchers limited potential for sample bias [28, 31]. Professional collaborators who agreed to advertise the survey included the European Agency for Safety and Health at Work (EU-OSHA), the University College Dublin Centre for Safety and Health at Work, and the British Columbia General Employees’ Union. The survey link was posted in 22 international, OSH-themed LinkedIn and Facebook groups accounting for 270,000 total group members.

#### Survey results

To assess associations between participant characteristics and workplace protection, odds ratios (OR) and adjusted odds ratios (AOR) generated from logistic regression models were used to estimate the likelihood of feeling protected at work vs. feeling unprotected or unsure according to age, gender, country of residence, education level, occupation, and vaccination status. Stepwise model selection by Akaike information criterion (AIC) was performed to determine the best-fit multivariable model using R version 4.0.2 step() function (R Foundation for Statistical Computing, Vienna, Austria). Survey data used for logistic regression is provided in Additional file 1.

### Phase 3. Psychometric property evaluation

Quantitative survey validation followed recommended guidelines for survey reliability and validity testing [32]. All data were analysed in R. Note that content validity was tested prior to data collection during the survey development phase. Survey reliability, criterion and construct validity were evaluated following data collection.

*Reliability:* Prior to data collection, the researchers hypothesized that preventive constructs fell under seven IPC measures’ domains: basic preventive measures, environmental adjustments, surveillance, contact tracing, PPE, education and training, and vaccination. To test this, exploratory factor analysis (EFA) was conducted using R package ‘psych’ 2.1.6 to identify the underlying factor structure explaining the relationship between 44 measured variables [33]. Questions relating to medical PPE and contact tracing were excluded from EFA due to large quantities of missing data. To determine the suitability of data for EFA, Pearson correlation matrices were verified for a statistically significant Bartlett’s test and a Kaiser-Meyer-Olkin (KMO) statistic above 0.60 [33]. Scree plot inspections and parallel analysis based on minimum rank factor analysis (PA-MRFA) were conducted to determine the advised number of factor dimensions. The PA-MRFA method, which expresses model fit by reporting the overall percentage of common variance explained [33], has been recommended for assessing the number of common factors underlying polytomous variables [34]. The Oblimin oblique rotation (non-orthogonal) method was used to aid factor interpretation, with rotated loadings above 0.32 considered acceptable [35]. Data and codes used for EFA, and a description of coded response options are available in Additional files 2, 3.

Cronbach’s alpha coefficients and values of alphas if removed were calculated to measure internal consistency within the IPC domains identified through EFA. Values ≥0.6 indicated acceptable internal consistency – that is, that all questions within the domain measured the appropriate protective construct – a threshold that has been recommended in the early stages of research [36, 37].

*Criterion Validity:* Criterion validity is the relation between the score of an instrument and, conventionally, another instrument that is widely accepted as a ‘gold standard’ [32]. Because this was the first survey intended to measure the level of COVID-19 protection in a participants’ workplace, no accepted instrument existed for comparison. Instead, researchers asked participants to identify whether or not they felt protected in the workplace at the end of the survey, determining whether investigated protective measures related to participants’ own standards for adequate safety. This method followed a similar procedure to Watkins et al. who looked for an alternative to a long instrument to assess depression and tested a single question – Do you frequently feel sad or depressed [38]? This study sought to verify a long instrument to assess COVID-19 workplace safety by comparing it to a single question – Do you feel protected from COVID-19 at work?

To test for criterion validity, an overall COVID-19 IPC measures’ protective score was calculated out of 40 possible points and compared to the survey’s ‘gold standard’ question. Respondents received one point for each affirmative response to questions on basic preventive measures, environmental adjustments, testing and surveillance, education and training, PPE, contact tracing, vaccination status, and access to paid sick leave. Detailed score calculations are provided in Additional file 4. Protective scores were compared to whether or not a worker felt protected from COVID-19 at work using two-sample t-tests; the significance level was set at *p* < 0.05.

*Construct Validity:* To understand the extent to which a set of variables represent the construct intended to be measured (i.e., construct validity), discriminant validity tests the hypothesis that a target-measurement is not improperly related to variables from which it should differ [39]. As the survey aimed to measure the level of occupational COVID-19 protection available to workers, a higher number of IPC measures in place should not be associated with feeling unprotected at work and vice versa. To test this, respondents were divided into quartiles according to total protective measures scores. Odds ratios (OR) generated from univariable logistic regression were used to estimate the likelihood of feeling protected at work according to protective score quartile. Known-groups technique was applied to assess whether the survey instrument successfully captured expected differences between groups of survey respondents. Hypotheses were formulated prior to data collection and tested using two-sample t-tests (significance level: *p* < 0.05).

## Results

### Participant characteristics and survey results

A total of 1473 eligible participants viewed and clicked on the survey link. Participants were given access to the study information sheet from the survey home page. Informed consent to participate was requested before the survey could begin. A total of 890 surveys were completed giving a response rate of 60.4%. The survey took 6.5 min to complete on average (min: 1.9 – max: 16.5). 70% (627/890) of respondents were actively in the workplace (i.e., not working from home or out of work) and included in psychometric property evaluation. A description of active workers’ socio-demographic and occupational characteristics is displayed in Table 1 along with univariable and multivariable logistic regression results for feeling protected at work vs. feeling unprotected or unsure.

**Table 1:** Participant characteristics and logistic regression results for feeling protected from COVID-19 at work vs. feeling unprotected or unsure (*N* = 627)

		N	%	Do you feel protected from COVID-19 at work? Yes vs. No or unsure	
				Crude OR (95% CI)	Adjusted OR (95% CI)^**c**^
**Gender**	Female^a^	436	71		
	Male	181	29	1.99 (1.35,2.94)***	1.88 (1.18,2.99)**
	Total	617	100		
**Age**	18 to 34^a^	151	24		
	35 to 44	165	26	1.05 (0.66,1.67)	1.29 (0.76,2.21)
	45 to 54	177	28	1.5 (0.94,2.40)	1.64 (0.98,2.77)
	55 and over	134	21	1.84 (1.10,3.05)*	2.17 (1.25,3.77)**
	Total	627	100		
**Country of Residence**^**b**^	Ireland ^a^	210	36		
	Argentina	24	4	2.02 (0.71,5.76)	
	Canada	243	42	0.80 (0.54,1.18)	
	China	26	4	2.15 (0.76,6.07)	
	Nigeria	18	3	1.16 (0.41,3.27)	
	Poland	16	3	1.05 (0.37,3.02)	
	UK	49	8	0.40 (0.20,0.78)**	
	Total	586	100		
**Education Level**	No Higher/Third Level Education ^a^	146	23		
	Higher/Third Level Education and up	481	77	0.71 (0.47,1.06)	
	Total	627	100		
**Occupational Sector**	Healthcare or social assistance^a^	255	50		
	Construction	15	3	1.18 (0.39,3.55)	0.98 (0.23,4.23)
	Educational services	106	21	0.48 (0.30,0.79)**	0.74 (0.26,2.13)
	Administration	19	4	0.65 (0.26,1.67)	2.03 (0.75,5.53)
	Manufacturing and food processing	15	3	7.64 (0.98,59.43)	6.58 (0.67,64.61)
	Professional, scientific, or technical services	31	6	1.85 (0.76,4.50)	2 (0.55,7.25)
	Public administration and defence	25	5	0.82 (0.35,1.93)	1.37 (0.37,5.04)
	Transportation or warehousing	17	3	0.46 (0.16,1.27)	0.44 (0.09,2.1)
	Retail trade	27	5	0.23 (0.09,0.57)**	0.38 (0.1,1.46)
	Total	509	100		
**Role**	Employee^a^	432	69		
	Management	195	31	2.99 (2.01,4.43)***	3.1 (1.99,4.83)***
	Total	627	100		
**Vaccination Status**	Vaccinated (Fully or Partially) ^a^	460	73		
	Unwilling to be vaccinated	62	10	0.82 (0.48,1.43)	
	No access to COVID vaccine	105	17	0.57 (0.34,0.95)*	
	Total	627	100		

*p < 0.05, **p < 0.01, ***p < 0.001

^a^ Reference category

^b^ Because this study aimed to determine the survey’s ability to capture country-level differences in response, we chose not to combine groups despite low percentages of responses from some countries (~ 3%)

^c^ Stepwise descending variable selection using AIC

Results from univariable logistic regression showed that men were more likely to feel protected at work than women (OR = 1.99; 95% CI = 1.35,2.94), and that older workers (> 55 yrs.) were more likely to feel protected at work than young workers (< 35 yrs.) (OR = 1.84. 95% CI = 1.10,3.05). Though 78% (453/586) of survey responses yielded from Ireland and Canada, responses from Argentina, China, Nigeria, Poland, and the UK were also collected. Respondents from the UK were significantly less likely to feel protected at work than Irish respondents (OR = 0.40; 95% CI =0.20,0.78). Half of survey respondents were healthcare workers (255/509); the remaining half yielded from diverse occupational sectors. Educators felt significantly less protected at work than healthcare workers (OR = 0.48; 95% CI = 0.30,0.79**)**, as did retail workers (OR = 0.23; 95% CI = 0.09,0.57). Managers, accounting for 31% of respondents (195/627), were significantly more likely to feel protected than employees (OR = 2.99; 95% CI = 2.01,4.43). Compared to the 73% (460/627) of vaccinated respondents, having no access to a COVID-19 vaccine left workers feeling unprotected (OR = 0.57; 95% CI =0.34,0.95). Choosing not to be vaccinated was not significantly associated with feeling unprotected at work (OR = 0.82; 95% CI = 0.48,1.43). In the adjusted model, men (AOR = 1.88; 95% CI = 1.18,2.99), over 55’s (AOR = 2.17; 95% CI = 1.25,3.77), and managers (AOR = 3.1; 95% CI = 1.99,4.83) remained significantly more likely to feel protected at work after adjustment for gender, age, professional role, and occupational sector.

**Fig. 3:**
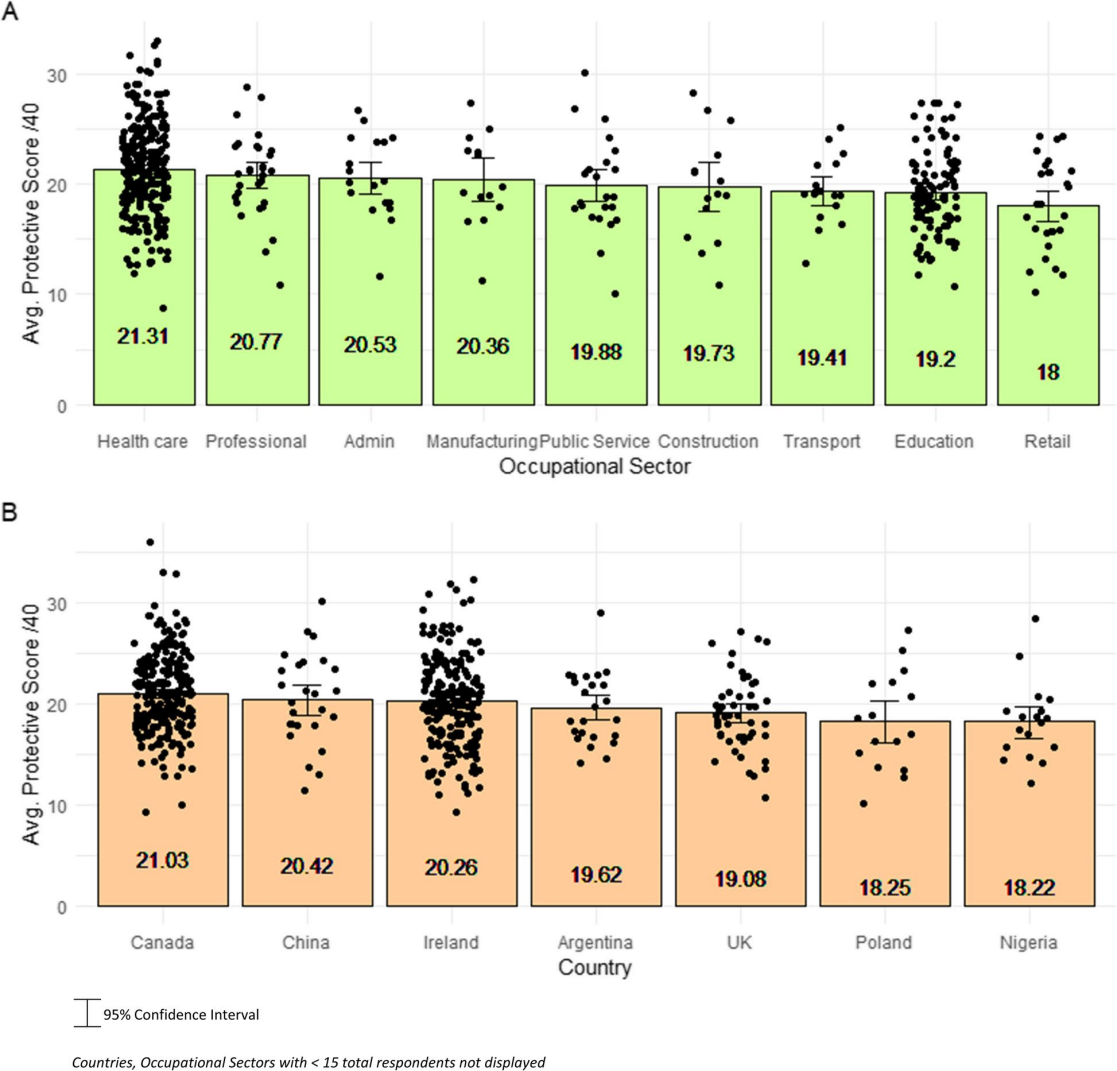
Average number of workplace protective measures identified by occupational sector (*N* = 509) (**A**) and country (*N* = 586) (**B**). *95% Confidence Intervals displayed. ** Countries, Occupational Sectors with < 15 total respondents not displayed

Protective measure scores out of 40 were calculated for all actively working respondents (*n* = 627) based on the number of safety measures identified in their workplace. Participants scored 21 points on average (min: 9 – max: 36). Average protective measures scores are displayed by country and occupational sector in Fig. 3.

### Survey validation results

Survey data was determined suitable for EFA with evidence of substantial correlations between items, Bartlett’s statistic = 7584.4 (*df* = 946), *p* < 0.001, and KMO = 0.77. Scree plots and PA-MRFA suggested a 9-factor model accounting for 67.25% of common variance explained. Items for which loadings were non-significant (< 0.32) were dropped from respective factors. Internal consistency results for the nine identified IPC measures survey domains are shown in Table 2. All domains satisfied internal reliability criteria (Cronbach α ≥ 0.60). Masking and social distancing domains had the highest average inter-item correlations (Cronbach α = 0.90, 0.97); domains relating to costs incurred and limiting contacts had the lowest (Cronbach α = 0.60). Questions on vaccination status, access to a contact tracing program, handwashing, and use of signage in the workplace did not fit into identified IPC domains but were kept as separate constructs to ensure content validity. Questions on the need to improve vaccination uptake amongst employees, the use of plastic barriers, and masking of non-employees were removed from the survey.

**Table 2:** Internal consistency of COVID-19 IPC Measures Survey Domains as indicated by Cronbach’s alpha (n = 627 actively working respondents)

COVID-19 IPC Measures Domain^a^	Items^b^	α if item removed^**c**^
**Areas for Improvement** α = 0.73 ^c^	Contact tracing	0.69
	Education and training	0.69
	Access to protective materials	0.69
	Testing	0.70
	Implementation of basic preventive measures by management	0.70
	Adherence to basic preventive measures by employees	0.70
	Funding for safety measures	0.72
	Environmental conditions	0.72
	Access to vaccines	0.73
**Environmental Adjustments** α = 0.66 ^c^	Temperature adjustments	0.56
	Ventilation adjustments	0.65
	Environmental monitoring	0.61
	Air quality monitoring	0.56
**Testing and Surveillance** α = 0.62 ^c^	Testing of symptomatic employees	0.48
	Testing of close contacts	0.51
	Employee temperature checks	0.62
	Symptom reporting to management	0.57
**Employee Education and Training** α = 0.68 ^c^	Training on proper PPE use	0.66
	Training on how to safely interact with colleagues in work	0.49
	Training on how to safely socialize outside of work	0.61
**Costs Incurred** α = 0.60 ^c^	Free COVID-19 testing	0.54
	Paid time off for testing	0.54
	Paid sick leave	0.54
**Number of Contacts** α = 0.60 ^c^	Worker movement restricted between facilities	0.45
	Worker bubbles	0.51
	Only essential personnel enter facility	0.57
	High-risk employees (> 60 yrs., underlying health conditions) stay at home	0.58
**Physical Distancing** α = 0.97 ^c^	Physical distancing rules in place	0.95
	Physical distancing is maintained	0.92
**Isolation Strategies** α = 0.73 ^c^	Confirmed COVID-19 cases self-isolate	0.56
	Symptomatic employees self-isolate	0.54
	Close contacts self-isolate	0.78
**Masking** α = 0.90 ^c^	All employees are instructed to wear masks	0.83
	Masks are worn correctly in the workplace	0.81

^a^Domains identified through exploratory factor analysis

^b^ Items coded as: Yes (2), Unsure (1), No (0)

^c^ Standardized alpha’s reported

Hypotheses defined prior to data collection and results from criterion and construct testing are displayed in Table 3. To assess criterion validity, protective measures’ scores were compared to the survey’s ‘gold-standard’ question: Do you feel protected from COVID-19 in the workplace? A significant association was identified using two-sample t-tests (*p* < 0.001), confirming a relation between a participants’ survey results and feeling protected at work. More precisely, to determine discriminate validity, participant protective scores were divided into quartiles ranging from highest protection (23–36 total measures in place) to lowest (≤ 17 total measures). Results from univariable logistic regression showed that participants in the highest protective quartile were significantly more likely to feel protected from COVID-19 at work than those in the lowest quartile (OR = 2.67; 95% CI = 1.17,2.95); and that participants in the lowest quartile were significantly less likely to feel protected than participants in the highest quartile (OR = 0.38; 95% CI = 0.23,0.62).

**Table 3:** Criterion and construct validity of COVID-19 IPC Measures Survey (n = 627 actively working respondents)

Validity Measure	Hypothesis tested	n	Test used	Result	Conclusion
Concurrent validity	There is a positive association between overall protective measures score and the ‘gold-standard’ question: Do you feel protected from COVID-19 at work?	583^a^	Two-sample t-test^b^	*p* < 0.001 ***	The survey instrument successfully measures the degree of COVID-19 safety a participant feels in the workplace.
Known-groups technique	The number of protective measures in place varies significantly by country	453^c^	Two-sample t-test^bc^	*p* = 0.04 *	The survey instrument successfully captures national differences in workplace protective response
Known-groups technique	The health care and social assistance sectors have more protective measures in place than other occupational sectors	627	Two-sample t-test^b^	*p* < 0.001***	The survey instrument successfully captures occupational differences in workplace protective response
Discriminant validity	A participant in the highest protective scores quartile (i.e., whose workplace has more protective measures in place than 75% of survey respondents) is significantly more likely to feel protected at work than a participant in the lowest protective scores quartile	583 ^a^	Univariable logistic regression^f^	OR (95% CI) = 2.67 (1.17,2.95)***	Respondents with high IPC coverage are more likely to feel protected at work than those with low IPC coverage. The survey instrument is not improperly measuring the level of protection available to workers.
Discriminant validity	A participant in the lowest protective scores quartile (i.e., whose workplace has fewer protective measures in place than 75% of survey respondents) is significantly less likely to feel protected at work than a participant in the highest protective scores quartile	583 ^a^	Univariable logistic regression^d^	OR (95% CI) = 0.38 (0.23,0.62)***	Respondents with low IPC coverage are less likely to feel protected at work than those with high IPC coverage. The survey instrument is not improperly measuring the level of protection available to workers.

*p < 0.05, **p < 0.01, ***p < 0.001

^a^ Data on ‘Do you feel protected from COVID-19 at work?’ missing for *n* = 44 participants

^b^ Heterogeneity of variances verified- Bartlett test; normality verified -Shapiro-Wilks statistic

^c^ Two largest participant groups compared: Canada (*n* = 243) and Ireland (*n* = 210)

^d^ Dependent variable: Feeling protected at work vs. feeling unprotected or unsure

Construct validity was assessed using known-groups technique, whereby expected differences between groups of respondents were tested and verified. Significant differences in average protective score were detected between Irish and Canadian respondents, the two largest participant groups, according to two-sample t-tests (*p* = 0.04). Health care and social assistance sectors had more protective measures in place than other occupational sectors (*p* < 0.001), as hypothesized. Results displayed in Table 2 further demonstrate the survey’s ability to distinguish between groups. Feeling protected varied significantly by country (i.e., UK respondents were less likely to feel protected at work than Irish respondents), and by occupational sector (i.e., retail workers and educators were less likely to feel protected than healthcare workers).

The final survey instrument based on pre-testing and psychometric property evaluation is available in Additional file 5.

## Discussion

This survey was developed to measure the level of occupational COVID-19 protection available to workers from diverse geographic and occupational settings. Following expert consultation and pre-testing, the survey showed strong face and content validity. Psychometric property evaluation revealed satisfactory levels of internal consistency reliability, and criterion and construct validity. The survey provides a framework for evaluating the level of protection a worker perceives they have from COVID-19 in the workplace. It holds the potential to be of particular use to decision-makers in the distribution of infectious disease prevention and control resources, and to guide recommendations for keeping the global workforce safe and healthy at the onset of emerging infectious disease outbreaks.

Exploratory factor analysis revealed nine key IPC domains that were confirmed by a high percentage of common variance explained. Cronbach’s alpha coefficients showed that items in each domain measured the appropriate construct. By providing a more precise model than originally hypothesized by the researchers, EFA provided insight into how occupational IPC guidelines and programs can be structured. For example, limiting worker movements and contacts emerged as a separate construct from physical distancing between workers. Testing and isolation measures emerged as separate from the cost of testing and isolation incurred. Survey items on universal testing, contact tracing, vaccinations, and signage did not fit into the 9-domain model. However – because research shows the ability of universal testing and timely contact tracing to prevent COVID-19 outbreaks [6]; that the rollout of vaccines plays a crucial part in protecting workers [40]; and that COVID-19 training should be reinforced by the use of signage placed in strategic locations [41] – each of these items were considered important for content validity and left in the survey.

By design, this study focused on survey development and validation. Nevertheless, survey results and hypothesis testing applied in the context of psychometric property evaluation revealed important considerations for occupational health. In terms of country-level differences, Canadian workers identified a higher number of implemented COVID-19 IPC precautions than Irish respondents despite representing a lower percentage of health care workers (42% of Canadian respondents worked in health care or social assistance vs. 60% of Irish respondents). This may be explained, in part, by an older sample of Canadian respondents. Research shows that older and more experienced workers report greater comfort with IPC skills [42], corroborating our finding that older workers were twice as likely to feel protected as younger workers. However, with both countries reporting similar vaccination rates and the implementation of return-to-work COVID-19 safety policies [9, 14], it will be important to examine survey data for specific variations in workplace IPC response. Though the total number of protective measures did not vary significantly between UK and Irish respondents, UK respondents reported feeling significantly less protected at work. One explanation relates to measures’ adherence. For example, over one third of UK respondents reported that colleagues ‘rarely’ or ‘never’ wore masks correctly, compared to just 10% of Irish respondents. This demonstrates how individuals may perceive and prioritize safety differently, particularly when COVID-19 safety precautions have the potential to create physical or social discomfort [9, 43], add to the workload [42], or result in a loss of income (32% of survey respondents reported not receiving paid time off to be tested for COVID-19). Consequently, voluntary adherence to instituted IPC measures will require clear and systematic communication on the necessity for and strategy in place behind them [44]. Though relatively few workers responded from developing countries, we chose to report findings from Nigeria and Poland to demonstrate the importance of targeting workers in less-studied, low- and middle-income countries in future survey distribution. Nigerian participants reported some of the lowest numbers of protective measures despite a surge in cases at the time of survey distribution, indicating that factors such as financial resources and public health infrastructure may be contributing to differences in occupational COVID-19 response.

Feeling protected at work varied according to gender, professional role, and occupational sector. In line with reports that women are more likely to feel concerned over their own health and the health of others’ [45], survey results indicated that men felt significantly more protected at work than women despite higher rates of vaccination amongst female workers (77% of women were vaccinated, compared to 66% of men). The COVID-19 pandemic has exacerbated gender inequalities. Despite being more likely to work on the frontline and in essential sectors, women are severely underrepresented in COVID-19 decision-making bodies [46]. We found that twice as many male respondents were in managerial roles as female respondents, and that managers felt more protected than employees after accounting for sector effect. Mediation analysis will be of value in the next phase of survey roll-out to assess the role of various factors on the pathway between gender and feeling protected from COVID-19 at work. Educators and retail workers felt significantly less protected than healthcare workers, with health care workers reporting the highest average protective score and retail workers the lowest. While IPC response has been widely studied in the healthcare setting [6], few studies have examined COVID-19 safety amongst retail, transport, and other essential workers. Our finding that 55% (197/359) of non-health care respondents felt unprotected at work, coupled with reports of COVID-19 outbreaks in international bars, building sites, food-processing plants, hotels, shops, and transportation settings [4], underline the value in studying the COVID-19 safety resources available to geographically and occupationally diverse groups of workers. By successfully capturing gender and occupational inequalities, the survey is an effective tool for tailoring disease prevention and control response to those most in need of protection.

Similar to findings from Ireland and Canada [47, 48], 10% of survey respondents reported an unwillingness to be vaccinated for COVID-19. This group did not feel significantly less protected at work than vaccinated participants. Misinformation is likely to be contributing to a sense of false security. Research shows that vaccine-hesitant individuals consume significantly less information about COVID-19 from newspapers, television, radio, and government agencies, reporting higher levels of trust in social media [49]. Recommendations for addressing vaccine hesitancy include collaborations between health agencies and multiple societal stakeholders to avoid the feeling that public health measures are for the benefit of government authorities [50]; and public health messaging that is targeted, clear, direct, repeated, positively orientated, and emphasises the personal benefits of vaccination against COVID-19 [49]. Similar recommendations can be applied in the workplace to ensure adequate awareness of and adherence to occupational safety precautions that are important in addition to vaccination campaigns. Of 456 vaccinated survey respondents, 38% still did not feel adequately protected from COVID-19 at work.

Pre and pilot testing resulted in additional lessons learned regarding survey distribution. The multi-modal recruitment strategy used resulted in greater uptake than reported in other, similar studies (McRobert et al. received 387 complete responses from a multidisciplinary international sample of clinicians using social media posts and email distribution over a three-month period [27]). Nevertheless, we found that posting in relevant occupational LinkedIn groups garnered little to no response. Posting in occupational Facebook groups was slightly more successful, however researchers were required to post from personal profiles creating a level of individual exposure not identified by other studies that used Facebook groups to recruit for health research [51, 52]. The most successful recruitment method involved partnering with relevant organizations who could distribute the survey link via their email network (e.g., EU-OSHA, UCD Centre for Safety and Health at Work, BC General Employees’ Union). However, ensuring partner participation required extensive effort. Of 132 emails sent by the researchers to international trade unions and occupational safety and health organizations, seven eventually agreed to distribute the survey (5%). Reasons for declined participation included precarious political and/or pandemic-related circumstances, overworked staff, and survey fatigue. As a next step, and to ensure as robust and unbiased a study sample as possible, the research team plans to roll out the survey in select target countries using Qualtrics^XM^ Research Services; to post the survey link to personal social media profiles and encourage snowballing with the aid of a promotional video (Additional file 7); and to continue working with external partners to solicit participation via email recruitment.

### Limitations

This study has several limitations. Because all survey data was collected anonymously via Qualtrics^XM^ survey platform, test-retest reliability could not be assessed. Despite efforts to gather pilot data from as representative a sample as possible, survey uptake was greater in Ireland and Canada than in other targeted countries. Workers in high-risk occupational settings like manufacturing and food-processing, retail, transport, and construction also proved difficult to recruit. Furthermore, while the researchers have developed strategies for a more equitable roll-out of the validated survey instrument moving forward, we feel it important to note the limited ability of an online survey instrument to capture viewpoints of potentially vulnerable workers with limited literacy skills and/or access to technology.

Though the survey was translated into multiple languages with the aid of key informants, allocated research funding did not allow for forward and back translation by two independent reviewers in each language nor expert committee review as recommended [24]. As well, most survey responses were based on the English version of the survey making psychometric property evaluation less relevant to the translated versions. Researchers or organizations should thus consider additional psychometric property evaluation of survey translations before distribution to a non-English speaking workforce. Despite these limitations, pilot data allowed for sufficient psychometric property evaluation in English.

## Conclusions

A multi-lingual online survey to measure the level of occupational COVID-19 protection available to workers in diverse geographic and occupational settings was developed, piloted, and validated. The survey showed strong face and content validity in English, as well as internal consistency, criterion validity, and construct validity. Translated versions of the survey into Polish, Spanish, French, Hindi, and Chinese demonstrated potential for adaptability across working cultures and countries. The survey instrument is suitable for measuring occupational COVID-19 IPC response in varied global and workplace settings, providing valuable insight for COVID-19 occupational safety and health guidelines and future infectious disease preparedness. As a follow-up to this research, survey data will be used to map and compare occupational COVID-19 IPC measures used in international workplace settings.

## Supplementary Information

**Additional file 1.**

**Additional file 2.**

**Additional file 3.**

**Additional file 4.**

**Additional file 5.**

**Additional file 6.**

**Additional file 7.**

## Data Availability

All data generated or analysed during this study are included in this published article and its supplementary information files. Translated versions of the survey are available upon request to the lead researcher at carolyn.ingram@ucd.ie.

## Abbreviations

AIC: Akaike information criterion
AOR: Adjusted odds ratio
BC: British Columbia
EFA: Exploratory factor analysis
EU-OSHA: European Agency for Safety and Health at Work
ILO: International Labour Organization
IPC: Infection prevention and control
KMO: Kaiser-Meyer-Olkin statistic
OR: Odds ratio
OSH: Occupational safety and health
OSHA: United States’ Occupational Safety and Health Administration
PA-MRFA: Parallel analysis - minimum rank factor analysis
PPE: Personal protective equipment
UCD: University College Dublin

## References

[1] Bi Q, Wu Y, Mei S, Ye C, Zou X, Zhang Z (2020). Epidemiology and transmission of COVID-19 in 391 cases and 1286 of their close contacts in Shenzhen, China: a retrospective cohort study. Lancet Infect Dis.

[2] Morawska L, Milton DK (2020). It is time to address airborne transmission of coronavirus disease 2019 (COVID-19). Clin Infect Dis.

[3] Occupational Safety and Health Administration (OSHA) (2020). Guidance on preparing workplaces for COVID-19.

[4] Leclerc QJ, Fuller NM, Knight LE, Funk S, Knight GM (2020). What settings have been linked to SARS-CoV-2 transmission clusters?. Wellcome Open Res.

[5] Greenhalgh T, Jimenez JL, Prather KA, Tufekci Z, Fisman D, Schooley R (2021). Ten scientific reasons in support of airborne transmission of SARS-CoV-2. Lancet.

[6] Ingram C, Downey V, Roe M, Chen Y, Archibald M, Kallas K-A (2021). COVID-19 prevention and control measures in workplace settings: a rapid review and meta-analysis. Int J Environ Res Public Health.

[7] Dehghani F, Omidi F, Yousefinejad S, Taheri E (2020). The hierarchy of preventive measures to protect workers against the COVID-19 pandemic: a review. Work..

[8] Hanke W, Pietrzak P (2021). Biological security of the SARS-CoV-2 (COVID-19) infection in large workplaces outside the healthcare sector – an epidemiologist’s point of view. Med Pr.

[9] International Labor Organization (ILO) (2021). Protecting the life and health of workers during the COVID-19 pandemic: overview of national legislative and policy responses.

[10] European Centre for Disease Prevention and Control (2021). Rapid risk assessment: COVID-19 outbreaks in long-term care facilities in the EU/EEA in the context of current vaccination coverage, 26 July 2021.

[11] Fegan C. More than one-third of Covid-19 cases in Ireland linked to workplaces. Independent. 2021; [cited 2021 Aug 16]; Available from: https://www.independent.ie/irish-news/health/more-than-one-third-of-covid-outbreaks-linked-to-workplaces-40754437.html.

[12] United Food and Commercial Workers (2021). UFCW: OSHA COVID workplace safety standard fails to protect frontline grocery and meatpacking workers still at risk from pandemic.

[13] OSHA (2021). Subpart U—COVID-19 healthcare ETS [internet]. Subpart U—COVID-19 healthcare emergency temporary standard.

[14] Johns Hopkins Coronavirus Resource Center (2020). COVID-19 map [internet].

[15] Centers for Disease Control and Prevention (2020). When You've been fully vaccinated [internet].

[16] Hsu A. Federal COVID Workplace Safety Rules Are Here. But Only For Health Care Workers. National Public Radio [Internet]. June 10 [cited 2021 Jun 15]. The coronavirus crisis. Available from: https://www.npr.org/2021/06/10/1005036698/federal-covid-workplace-safety-rules-finally-here-but-only-for-health-care-worke. 2021

[17] European Union (2021). Re-open EU.

[18] Smith K, Ramsay C. State of local emergency declared in Regional Municipality of Wood Buffalo amid high COVID-19 cases. Global News [Internet]. 2021 April 26 [cited 2021 Aug 12]; Health. Available from: https://globalnews.ca/news/7800424/state-of-local-emergency-to-be-declared-in-regional-municipality-of-wood-buffalo/

[19] Purnell N. India’s Covid-19 crisis tests the World’s Back offices. Wall Street J. 2021; May 22 [cited 2021 Sep 28]; Available from: https://www.wsj.com/articles/indias-covid-19-crisis-tests-the-worlds-back-offices-11621656009.

[20] Semple S, Cherrie JW (2020). Covid-19: protecting worker health. Ann Work Expo Health.

[21] Spinazzè A, Cattaneo A, Cavallo DM (2020). COVID-19 outbreak in Italy: protecting worker health and the response of the Italian industrial hygienists association. Ann Work Expo Health.

[22] ILO (2021). Country policy responses.

[23] Ananda-Rajah M, Veness B, Berkovic D, Parker C, Kelly G, Ayton D. Hearing the voices of Australian healthcare workers during the COVID-19 pandemic. BMJ Lead. 2021;5(1) [cited 2021 Sep 14]. Available from: https://bmjleader.bmj.com/content/5/1/31.

[24] Beaton DE, Bombardier C, Guillemin F, Ferraz MB (2000). Guidelines for the process of cross-cultural adaptation of self-report measures. Spine..

[25] Taylor RM, Crichton N, Moult B, Gibson F (2015). A prospective observational study of machine translation software to overcome the challenge of including ethnic diversity in healthcare research. Nurs Open.

[26] Miller JM, Harvey EM, Bedrick S, Mohan P, Calhoun E. Simple patient care instructions translate best: safety guidelines for physician use of Google translate. J Clin Outcomes Manag [Internet]. 2018;25(1) [cited 2021 Nov 18]. Available from: https://arizona.pure.elsevier.com/en/publications/simple-patient-care-instructions-translate-best-safety-guidelines.

[27] McRobert CJ, Hill JC, Smale T, Hay EM, Van der Windt DA. A multi-modal recruitment strategy using social media and internet-mediated methods to recruit a multidisciplinary, international sample of clinicians to an online research study. PLoS One. 2018;13(7):e0200184. 10.1371/journal.pone.0200184 PMC6034855 29979769

[28] Ball HL (2019). Conducting online surveys. J Hum Lact.

[29] Wejnert C, Heckathorn DD (2008). Web-based network sampling: efficiency and efficacy of respondent-driven sampling for online research. Sociol Methods Res.

[30] Hlatshwako TG, Shah SJ, Kosana P, Adebayo E, Hendriks J, Larsson EC (2021). Online health survey research during COVID-19. Lancet Digit Health.

[31] Alessi E (2010). Conducting an internet-based survey: benefits, pitfalls, and lessons learned. Soc Work Res.

[32] de Souza AC, Alexandre NMC, de Brito Guirardello E (2017). Psychometric properties in instruments evaluation of reliability and validity. Epidemiol E Serviços Saúde.

[33] Baglin J (2014). Improving your exploratory factor analysis for ordinal data: a demonstration using FACTOR. PARE..

[34] Timmerman ME, Lorenzo-Seva U (2011). Dimensionality assessment of ordered polytomous items with parallel analysis. Psychol Methods.

[35] Tabachnick BG, Fidell LS, Ullman JB (2019). Using multivariate statistics.

[36] Streiner DL (2003). Starting at the beginning: an introduction to coefficient alpha and internal consistency. J Pers Assess.

[37] Ursachi G, Horodnic IA, Zait A (2015). How reliable are measurement scales? External factors with indirect influence on reliability estimators. Procedia Econ Finance.

[38] Watkins C, Daniels L, Jack C, Dickinson H, van den Broek M (2001). Accuracy of a single question in screening for depression in a cohort of patients after stroke: comparative study. BMJ..

[39] Polit DF (2015). Assessing measurement in health: beyond reliability and validity. Int J Nurs Stud.

[40] World Health Organization, ILO (2021). Policy brief: preventing and mitigating COVID-19 at work.

[41] Department of Health and Human Services (2020). Strategies to reduce COVID-19 transmission at the Smithfield Sioux Falls pork plant.

[42] Silverberg SL, Puchalski Ritchie LM, Gobat N, Murthy S (2021). COVID-19 infection prevention and control procedures and institutional trust: perceptions of Canadian intensive care and emergency department nurses. Can J Anesth Can Anesth.

[43] Harvard Medical School (2020). Coping with face mask discomfort.

[44] Iftekhar EN, Priesemann V, Balling R, Bauer S, Beutels P, Valdez AC, et al. A look into the future of the COVID-19 pandemic in Europe: an expert consultation. Lancet Reg Health – Eur. 2021;0(0) [cited 2021 Aug 16]. Available from: https://www.thelancet.com/journals/lanepe/article/PIIS2666-7762(21)00162-9/abstract. 10.1016/j.lanepe.2021.100185 PMC8321710 34345876

[45] Central Statistics Office (2020). Social impact of COVID-19 on women and men.

[46] European Commission (2021). COVID-19 pandemic is a major challenge for gender equality.

[47] MacDonald NE (2015). Vaccine hesitancy: definition, scope and determinants. Vaccine..

[48] Dzieciolowska S, Hamel D, Gadio S, Dionne M, Gagnon D, Robitaille L (2021). Covid-19 vaccine acceptance, hesitancy, and refusal among Canadian healthcare workers: a multicenter survey. Am J Infect Control.

[49] Murphy J, Vallières F, Bentall RP, Shevlin M, McBride O, Hartman TK (2021). Psychological characteristics associated with COVID-19 vaccine hesitancy and resistance in Ireland and the United Kingdom. Nat Commun.

[50] Ruijs WLM, Hautvast JLA, Kerrar S, van der Velden K, Hulscher MEJL (2013). The role of religious leaders in promoting acceptance of vaccination within a minority group: a qualitative study. BMC Public Health.

[51] Thornton L, Batterham PJ, Fassnacht DB, Kay-Lambkin F, Calear AL, Hunt S (2016). Recruiting for health, medical or psychosocial research using Facebook: systematic review. Internet Interv.

[52] Davies B, Kotter M (2018). Lessons from recruitment to an internet-based survey for degenerative cervical myelopathy: comparison of free and fee-based methods. JMIR Res Protoc.

