# Supplementary material for: Development and validation of a multi-lingual online questionnaire for surveying the COVID-19 prevention and control measures used in global workplaces
Source: BMC Public Health. 2022 Jan 12;22:74. doi: 10.1186/s12889-022-12500-w (PMC8753024; doi:10.1186/s12889-022-12500-w)
Supplement: Supplementary file 3 — Additional file 3. [file 12889_2022_12500_MOESM3_ESM.pdf]

Psychometric Evaluation Results

Carolyn Ingram

8/30/2021

Exploratory Factor Analysis

Factorability of the Data

Kaiser-Meyer-Olkin statistic (KMO) = 0.77

Bartlett's test of sphericity statistic p < 0.001

Tests indicate the suitability of data for EFA.

Polychoric correlation matrices were tested due to the ordinal nature of data, but did not meet the KMO statistic requirement. Pearson correlations were used.

```
KMO(r=cor(efa))
```

```
## Kaiser-Meyer-Olkin factor adequacy
## Call: KMO(r = cor(efa))
## Overall MSA =  0.77
## MSA for each item = 
##             Handwashing             desinfection                 barriers 
##                    0.88                     0.89                     0.88 
##              distancing                 masking1                 masking2 
##                    0.68                     0.68                     0.87 
##                movement                  bubbles           restrictedentry 
##                    0.89                     0.85                     0.91 
##                    risk      distance_maintained          mask_maintained 
##                    0.85                     0.69                     0.68 
##             temperature              ventilation            env_monitoring 
##                    0.79                     0.81                     0.70 
##             air_quality                test_symp            test_contacts 
##                    0.71                     0.73                     0.72 
##                test_all              test_random           isolate_contact 
##                    0.69                     0.69                     0.84 
##        isolate_symptoms         isolate_positive               tem_checks 
##                    0.76                     0.74                     0.77 
##          symp_reporting       cost_test_combined  paidtime_test_combined 
##                    0.90                     0.77                     0.76 
##  paidtime_sick_combined               ct_program               ed_signage 
##                    0.74                     0.81                     0.80 
##            training_ppe     training_colleagues   training_socializeout 
##                    0.79                     0.82                     0.80 
##           improve_basic     improve_access_basic  improve_access_funding 
##                    0.78                     0.76                     0.75 
## improve_adherence_basic        improve_education               improve_ct 
##                    0.83                     0.79                     0.75 
##         improve_testing   improve_vaccine_access  improve_vaccine_uptake 
##                    0.74                     0.67                     0.65 
##             improve_env                  vaccine 
##                    0.82                     0.72
```

```
cortest.bartlett(efa)
```

```
## R was not square, finding R from data
```

```
## $chisq
## [1] 7584.387
##
## $p.value
## [1] 0
##
## $df
## [1] 946
```

Scree Plot and Parallel Analysis

You can also embed plots, for example:

```
fa.parallel(efa,fm="minrank", n.iter = 500)
```

```
## Loading required namespace: Rcsdp
```

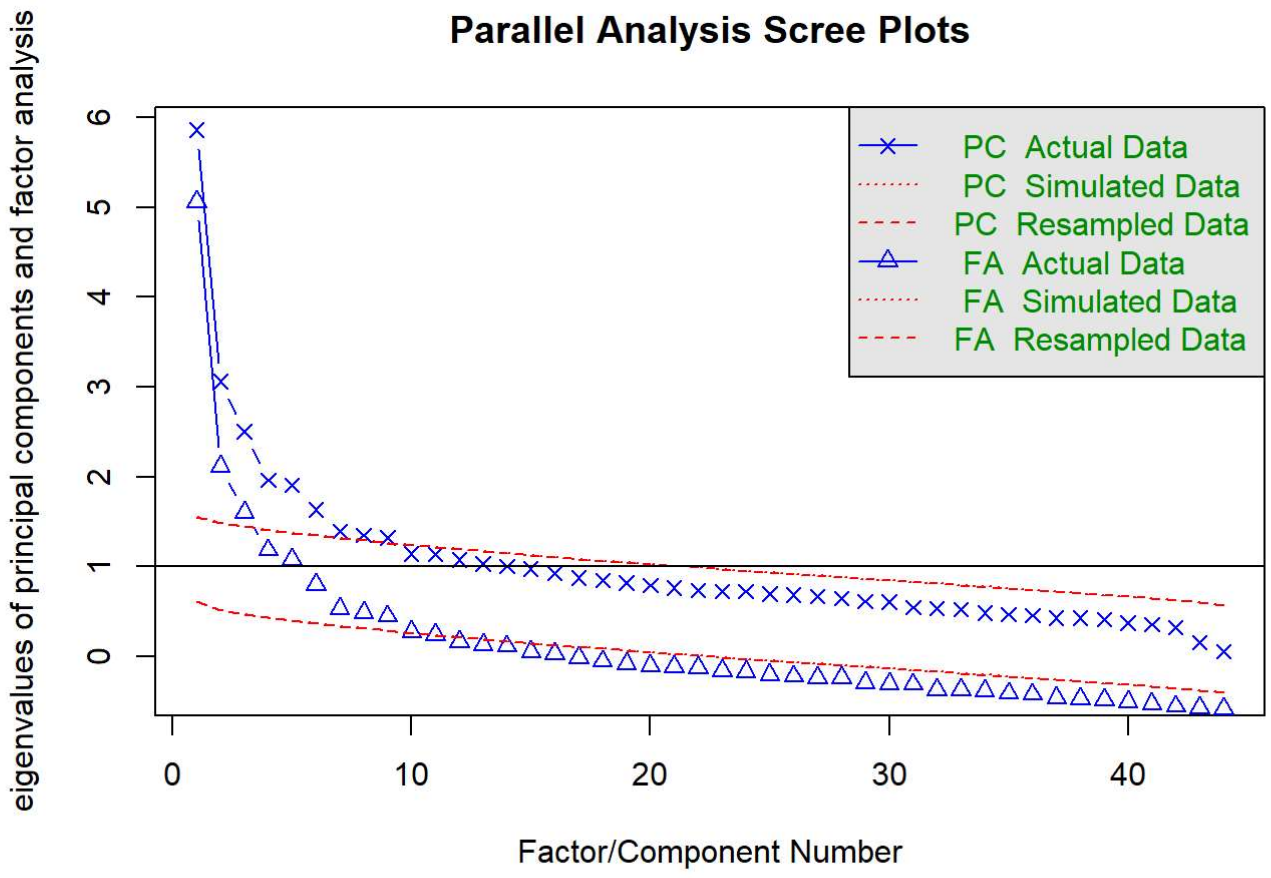


```
## Parallel analysis suggests that the number of factors =  9  and the number of components =  9
```

```
fa.nine <- fa(r=efa,
              nfactors = 9,
              covar = FALSE, SMC = TRUE,
              fm="minrank", # type of factor analysis we want to use (“minrank” is minimum rank
 factor analysis)
              max.iter=100, # (50 is the default, but we have changed it to 100
              rotate="oblimin") # none rotation
```

```
## Loading required namespace: GPArotation
```

```
print(fa.nine)
```

```
## Factor Analysis using method =  minrank
## Call: fa(r = efa, nfactors = 9, rotate = "oblimin", SMC = TRUE, covar = FALSE,
##     max.iter = 100, fm = "minrank")
## Standardized loadings (pattern matrix) based upon correlation matrix
##                          MRFA2 MRFA4 MRFA5 MRFA3 MRFA1 MRFA8 MRFA6 MRFA7 MRFA9
## Handwashing               0.03  0.14  0.16  0.18  0.13 -0.04  0.00 -0.04 -0.05
## desinfection             -0.12  0.23  0.11  0.17  0.14  0.10  0.15 -0.05 -0.15
## barriers                  0.01  0.41 -0.06  0.07  0.08  0.14  0.11 -0.09  0.09
## distancing                0.02  0.96  0.02 -0.01  0.00 -0.04  0.00  0.01  0.00
## masking1                  0.01 -0.01  0.86 -0.03  0.04  0.01 -0.01  0.04 -0.01
## masking2                  0.07  0.25  0.48  0.08 -0.13  0.09 -0.07 -0.05  0.17
## movement                 -0.02  0.21  0.17  0.11  0.16  0.09  0.05  0.06  0.37
## bubbles                  -0.07  0.23  0.00  0.15  0.14  0.15 -0.05 -0.10  0.35
## restrictedentry          -0.11  0.01  0.16  0.11  0.20  0.07  0.10 -0.04  0.25
## risk                      0.04  0.21  0.11  0.01  0.05  0.06  0.12  0.09  0.26
## distance_maintained      -0.02  0.93  0.00  0.01 -0.01  0.01  0.02  0.00  0.00
## mask_maintained          -0.04  0.00  0.97 -0.01 -0.02 -0.01  0.00  0.00 -0.01
## temperature              -0.09  0.02  0.07 -0.13  0.23  0.51 -0.13  0.17 -0.03
## ventilation              -0.08  0.06  0.07 -0.06  0.23  0.34 -0.13  0.00  0.15
## env_monitoring            0.07 -0.02  0.01 -0.05 -0.05  0.72  0.08  0.03  0.05
## air_quality               0.00 -0.03 -0.02  0.06 -0.04  0.78  0.00 -0.03 -0.06
## test_symp                 0.05  0.00  0.08  0.08 -0.07  0.02  0.14  0.59 -0.03
## test_contacts            -0.02 -0.01  0.01  0.18 -0.09  0.05  0.04  0.71 -0.01
## test_all                 -0.01 -0.18  0.00 -0.14  0.31  0.02  0.08  0.09  0.27
## test_random               0.03  0.05  0.02  0.01  0.08  0.04 -0.10  0.17  0.09
## isolate_contact          -0.10  0.01 -0.03  0.55  0.06  0.19 -0.03  0.02  0.12
## isolate_symptoms         -0.05  0.05 -0.03  0.77  0.02 -0.02 -0.04  0.05  0.01
## isolate_positive          0.04 -0.03  0.00  0.78  0.00 -0.07 -0.01  0.10 -0.03
## tem_checks                0.04  0.06  0.06 -0.10  0.36  0.01 -0.14  0.47  0.06
## symp_reporting           -0.01  0.13  0.03  0.16  0.15 -0.05  0.14  0.33  0.09
## cost_test_combined        0.00 -0.01 -0.01  0.01  0.06 -0.06  0.51  0.14  0.07
## paidtime_test_combined   -0.06  0.04 -0.02 -0.08  0.04  0.02  0.66  0.04  0.10
## paidtime_sick_combined   -0.03  0.15 -0.09 -0.08  0.03  0.05  0.57  0.06 -0.15
## ct_program               -0.04 -0.08  0.24  0.04  0.11  0.08  0.33  0.05  0.11
## ed_signage                0.12  0.12  0.09  0.23  0.01  0.04  0.30 -0.10 -0.20
## training_ppe              0.04  0.00  0.14  0.06  0.62 -0.09  0.05  0.08 -0.23
## training_colleagues      -0.02  0.10  0.01  0.10  0.65  0.06  0.09 -0.16 -0.01
## training_socializeout    -0.01 -0.01 -0.07 -0.02  0.65  0.04  0.03  0.02  0.19
## improve_basic             0.54 -0.09 -0.01 -0.02 -0.03 -0.03  0.09 -0.06  0.23
## improve_access_basic      0.58 -0.11  0.04  0.02 -0.09 -0.05  0.04 -0.06  0.29
## improve_access_funding    0.41 -0.08 -0.01  0.06 -0.06  0.06  0.00  0.00  0.10
## improve_adherence_basic   0.52 -0.10 -0.05  0.06  0.05 -0.03  0.05 -0.05 -0.04
## improve_education         0.62  0.02 -0.08 -0.02  0.05 -0.06 -0.03  0.07  0.02
## improve_ct                0.68  0.11 -0.06 -0.12  0.03  0.06 -0.07  0.07 -0.20
## improve_testing           0.58  0.02  0.08  0.03 -0.01  0.10 -0.12 -0.01 -0.18
## improve_vaccine_access    0.33  0.04  0.03  0.03  0.15  0.09 -0.15 -0.03  0.11
## improve_vaccine_uptake    0.21 -0.07 -0.02  0.15  0.14 -0.03  0.01 -0.04 -0.17
## improve_env               0.39  0.01  0.11  0.05 -0.06 -0.20  0.12 -0.04  0.02
## vaccine                   0.11 -0.09  0.14  0.26  0.09  0.01  0.33 -0.13 -0.30
##                             h2    u2 com
## Handwashing              0.138 0.862 4.3
## desinfection             0.288 0.712 6.4
## barriers                 0.278 0.722 1.9
## distancing               0.910 0.090 1.0
## masking1                 0.760 0.240 1.0
## masking2                 0.404 0.596 2.3
## movement                 0.394 0.606 3.1
## bubbles                  0.329 0.671 3.4
## restrictedentry          0.230 0.770 4.4
## risk                     0.211 0.789 3.3
## distance_maintained      0.885 0.115 1.0
## mask_maintained          0.917 0.083 1.0
## temperature              0.485 0.515 2.1
## ventilation              0.309 0.691 2.9
## env_monitoring           0.514 0.486 1.1
## air_quality              0.568 0.432 1.0
## test_symp                0.423 0.577 1.3
## test_contacts            0.585 0.415 1.2
## test_all                 0.227 0.773 3.4
```

```
## test_random             0.066 0.934 3.2
## isolate_contact         0.387 0.613 1.5
## isolate_symptoms        0.615 0.385 1.0
## isolate_positive        0.641 0.359 1.1
## tem_checks              0.422 0.578 2.3
## symp_reporting          0.296 0.704 3.0
## cost_test_combined      0.310 0.690 1.3
## paidtime_test_combined  0.456 0.544 1.1
## paidtime_sick_combined  0.388 0.612 1.5
## ct_program              0.253 0.747 2.8
## ed_signage              0.278 0.722 4.1
## training_ppe            0.500 0.500 1.5
## training_colleagues     0.534 0.466 1.3
## training_socializeout   0.488 0.512 1.2
## improve_basic           0.360 0.640 1.5
## improve_access_basic    0.453 0.547 1.7
## improve_access_funding  0.189 0.811 1.3
## improve_adherence_basic 0.304 0.696 1.2
## improve_education       0.398 0.602 1.1
## improve_ct              0.535 0.465 1.4
## improve_testing         0.394 0.606 1.4
## improve_vaccine_access  0.165 0.835 2.4
## improve_vaccine_uptake  0.117 0.883 4.1
## improve_env             0.234 0.766 2.0
## vaccine                 0.361 0.639 4.2
##
##                        MRFA2 MRFA4 MRFA5 MRFA3 MRFA1 MRFA8 MRFA6 MRFA7 MRFA9
## SS loadings             2.69  2.62  2.33  2.07  2.06  1.87  1.70  1.48  1.17
## Proportion Var          0.06  0.06  0.05  0.05  0.05  0.04  0.04  0.03  0.03
## Cumulative Var          0.06  0.12  0.17  0.22  0.27  0.31  0.35  0.38  0.41
## Proportion Explained    0.15  0.15  0.13  0.12  0.11  0.10  0.09  0.08  0.06
## Cumulative Proportion   0.15  0.30  0.42  0.54  0.65  0.76  0.85  0.94  1.00
##
##  With factor correlations of
##       MRFA2 MRFA4 MRFA5 MRFA3 MRFA1 MRFA8 MRFA6 MRFA7 MRFA9
## MRFA2  1.00 -0.12 -0.02 -0.05 -0.08 -0.10 -0.02 -0.03 -0.08
## MRFA4 -0.12  1.00  0.27  0.19  0.24  0.19  0.14  0.06  0.03
## MRFA5 -0.02  0.27  1.00  0.17  0.20  0.13  0.06  0.18  0.04
## MRFA3 -0.05  0.19  0.17  1.00  0.10  0.00  0.21  0.18 -0.02
## MRFA1 -0.08  0.24  0.20  0.10  1.00  0.23  0.17  0.15  0.08
## MRFA8 -0.10  0.19  0.13  0.00  0.23  1.00 -0.02  0.13  0.13
## MRFA6 -0.02  0.14  0.06  0.21  0.17 -0.02  1.00  0.04 -0.02
## MRFA7 -0.03  0.06  0.18  0.18  0.15  0.13  0.04  1.00  0.00
## MRFA9 -0.08  0.03  0.04 -0.02  0.08  0.13 -0.02  0.00  1.00
##
## Mean item complexity =  2.2
## Test of the hypothesis that 9 factors are sufficient.
##
## The degrees of freedom for the null model are  946  and the objective function was  12.42 wit
h Chi Square of  7584.39
## The degrees of freedom for the model are 586  and the objective function was  2.18
##
## The root mean square of the residuals (RMSR) is  0.03
## The df corrected root mean square of the residuals is  0.04
##
## The harmonic number of observations is  627 with the empirical chi square  1215.71  with prob
<  2.8e-46
## The total number of observations was  627  with Likelihood Chi Square =  1317.33  with prob <
3.5e-58
##
## Tucker Lewis Index of factoring reliability =  0.82
## RMSEA index =  0.045  and the 90 % confidence intervals are  0.041 0.048
## BIC =  -2457.06
## Fit based upon off diagonal values = 0.95
## Measures of factor score adequacy
##                                                   MRFA2 MRFA4 MRFA5 MRFA3 MRFA1
## Correlation of (regression) scores with factors    0.94  0.99  0.98  0.93  0.92
## Multiple R square of scores with factors           0.88  0.97  0.97  0.87  0.84
## Minimum correlation of possible factor scores      0.76  0.94  0.93  0.73  0.68
##                                                   MRFA8 MRFA6 MRFA7 MRFA9
```

```
## Correlation of (regression) scores with factors    0.93  0.89   0.9  0.85
## Multiple R square of scores with factors           0.86  0.79   0.8  0.73
## Minimum correlation of possible factor scores      0.73  0.57   0.6  0.46
```

Internal Consistency Reliability

Nine domains identified through EFA-MRFA.

Internal Consistency - Environmental Adjustments

```
env <- efa %>% dplyr::select(temperature:air_quality)
psych::alpha(env, title = "env", check.keys = TRUE)
```

```
##
## Reliability analysis  env
## Call: psych::alpha(x = env, title = "env", check.keys = TRUE)
##
##   raw_alpha std.alpha G6(smc) average_r S/N   ase mean   sd median_r
##       0.65      0.66    0.62      0.33   2 0.023  1.5 0.51     0.35
##
##  lower alpha upper     95% confidence boundaries
## 0.61 0.65 0.69
##
##  Reliability if an item is dropped:
##                raw_alpha std.alpha G6(smc) average_r S/N alpha se  var.r med.r
## temperature         0.53      0.56    0.48      0.30 1.3    0.033 0.0225  0.28
## ventilation         0.64      0.65    0.56      0.38 1.9    0.025 0.0037  0.35
## env_monitoring      0.60      0.61    0.51      0.34 1.5    0.027 0.0031  0.35
## air_quality         0.56      0.56    0.48      0.30 1.3    0.030 0.0156  0.34
##
##  Item statistics
##                  n raw.r std.r r.cor r.drop mean   sd
## temperature    627  0.76  0.74  0.61   0.50  1.6 0.76
## ventilation    627  0.70  0.65  0.45   0.37  1.8 0.85
## env_monitoring 627  0.65  0.69  0.54   0.40  1.4 0.63
## air_quality    627  0.70  0.74  0.61   0.48  1.4 0.62
##
## Non missing response frequency for each item
##                   1    2    3 miss
## temperature    0.60 0.23 0.17    0
## ventilation    0.49 0.23 0.28    0
## env_monitoring 0.69 0.23 0.08    0
## air_quality    0.70 0.23 0.08    0
```

Internal Consistency - Testing and Surveillance

```
testsurv <- efa %>% dplyr::select(test_symp, test_contacts, tem_checks, symp_reporting)
psych::alpha(testsurv, title = "testsurv")
```

```
##
## Reliability analysis  testsurv
## Call: psych::alpha(x = testsurv, title = "testsurv")
##
##   raw_alpha std.alpha G6(smc) average_r S/N   ase mean   sd median_r
##       0.62      0.62    0.58      0.29 1.6 0.025  1.8 0.66     0.26
##
##  lower alpha upper     95% confidence boundaries
## 0.57 0.62 0.67
##
##  Reliability if an item is dropped:
##                raw_alpha std.alpha G6(smc) average_r  S/N alpha se  var.r med.r
## test_symp           0.52      0.51    0.42      0.26 1.06    0.034 0.0008  0.27
## test_contacts       0.48      0.48    0.38      0.24 0.92    0.036 0.0026  0.26
## tem_checks          0.62      0.62    0.55      0.36 1.66    0.026 0.0216  0.28
## symp_reporting      0.58      0.57    0.51      0.31 1.35    0.030 0.0353  0.23
##
##  Item statistics
##                  n raw.r std.r r.cor r.drop mean   sd
## test_symp      627  0.72  0.72  0.60   0.45  1.8 0.98
## test_contacts  627  0.74  0.74  0.65   0.50  1.7 0.96
## tem_checks     627  0.61  0.61  0.37   0.30  1.8 0.95
## symp_reporting 627  0.67  0.66  0.46   0.37  2.1 0.98
##
## Non missing response frequency for each item
##                   1    2    3 miss
## test_symp      0.59 0.01 0.40    0
## test_contacts  0.62 0.01 0.37    0
## tem_checks     0.59 0.04 0.37    0
## symp_reporting 0.44 0.04 0.51    0
```

Internal Consistency - Education and Training

```
edu <- efa %>% dplyr::select(training_ppe, training_colleagues, training_socializeout)
psych::alpha(edu, title = "edu", check.keys = TRUE)
```

```
##
## Reliability analysis  edu
## Call: psych::alpha(x = edu, title = "edu", check.keys = TRUE)
##
##   raw_alpha std.alpha G6(smc) average_r S/N   ase mean   sd median_r
##       0.68      0.68     0.6      0.42 2.1 0.022  1.8 0.72     0.44
##
##  lower alpha upper     95% confidence boundaries
## 0.63 0.68 0.72
##
##  Reliability if an item is dropped:
##                       raw_alpha std.alpha G6(smc) average_r  S/N alpha se var.r
## training_ppe               0.64      0.66    0.49      0.49 1.91    0.027    NA
## training_colleagues        0.47      0.49    0.32      0.32 0.95    0.040    NA
## training_socializeout      0.61      0.61    0.44      0.44 1.56    0.031    NA
##                       med.r
## training_ppe           0.49
## training_colleagues    0.32
## training_socializeout  0.44
##
##  Item statistics
##                         n raw.r std.r r.cor r.drop mean   sd
## training_ppe          627  0.78  0.75  0.53   0.45  2.1 1.00
## training_colleagues   627  0.83  0.82  0.69   0.56  1.8 0.99
## training_socializeout 627  0.73  0.77  0.59   0.48  1.4 0.77
##
## Non missing response frequency for each item
##                          1    3 miss
## training_ppe          0.47 0.53    0
## training_colleagues   0.58 0.42    0
## training_socializeout 0.82 0.18    0
```

Internal Consistency - Areas for Improvement

```
imp <- efa %>% dplyr::select(improve_basic:improve_vaccine_access, improve_env)
psych::alpha(imp, title = "imp", check.keys = TRUE)
```

```
##
## Reliability analysis  imp
## Call: psych::alpha(x = imp, title = "imp", check.keys = TRUE)
##
##   raw_alpha std.alpha G6(smc) average_r S/N   ase mean   sd median_r
##       0.73      0.73    0.72      0.23 2.7 0.016  1.4 0.45     0.22
##
##  lower alpha upper     95% confidence boundaries
## 0.7 0.73 0.76
##
##  Reliability if an item is dropped:
##                         raw_alpha std.alpha G6(smc) average_r S/N alpha se
## improve_basic                0.70      0.70    0.69      0.23 2.3    0.018
## improve_access_basic         0.70      0.69    0.68      0.22 2.3    0.018
## improve_access_funding       0.72      0.72    0.71      0.24 2.6    0.017
## improve_adherence_basic      0.70      0.70    0.69      0.23 2.4    0.018
## improve_education            0.69      0.69    0.68      0.22 2.2    0.019
## improve_ct                   0.69      0.69    0.68      0.22 2.2    0.019
## improve_testing              0.70      0.70    0.69      0.23 2.3    0.018
## improve_vaccine_access       0.73      0.73    0.72      0.26 2.8    0.016
## improve_env                  0.72      0.72    0.71      0.24 2.6    0.016
##                          var.r med.r
## improve_basic           0.0078  0.22
## improve_access_basic    0.0076  0.21
## improve_access_funding  0.0082  0.23
## improve_adherence_basic 0.0086  0.21
## improve_education       0.0079  0.21
## improve_ct              0.0076  0.21
## improve_testing         0.0089  0.22
## improve_vaccine_access  0.0064  0.25
## improve_env             0.0093  0.25
##
##  Item statistics
##                           n raw.r std.r r.cor r.drop mean   sd
## improve_basic           627  0.59  0.59  0.52   0.43  1.4 0.80
## improve_access_basic    627  0.60  0.62  0.56   0.47  1.3 0.73
## improve_access_funding  627  0.47  0.49  0.37   0.32  1.3 0.71
## improve_adherence_basic 627  0.60  0.58  0.50   0.43  1.5 0.89
## improve_education       627  0.65  0.64  0.58   0.50  1.5 0.85
## improve_ct              627  0.65  0.65  0.60   0.51  1.4 0.78
## improve_testing         627  0.60  0.59  0.52   0.44  1.5 0.86
## improve_vaccine_access  627  0.39  0.42  0.28   0.24  1.3 0.67
## improve_env             627  0.51  0.49  0.37   0.32  1.6 0.90
##
## Non missing response frequency for each item
##                            1    3 miss
## improve_basic           0.80 0.20    0
## improve_access_basic    0.84 0.16    0
## improve_access_funding  0.85 0.15    0
## improve_adherence_basic 0.73 0.27    0
## improve_education       0.76 0.24    0
## improve_ct              0.81 0.19    0
## improve_testing         0.75 0.25    0
## improve_vaccine_access  0.87 0.13    0
## improve_env             0.71 0.29    0
```

Internal Consistency - Physical Distancing

```
distance <- efa %>% dplyr::select(distancing, distance_maintained)
psych::alpha(distance, title = "distance", check.keys = TRUE)
```

```
##
## Reliability analysis  distance
## Call: psych::alpha(x = distance, title = "distance", check.keys = TRUE)
##
##   raw_alpha std.alpha G6(smc) average_r S/N    ase mean   sd median_r
##       0.97      0.97    0.94      0.94  30 0.0026  2.2 0.94     0.94
##
##  lower alpha upper     95% confidence boundaries
## 0.96 0.97 0.97
##
##  Reliability if an item is dropped:
##                     raw_alpha std.alpha G6(smc) average_r S/N alpha se var.r
## distancing               0.95      0.94    0.88      0.94  15       NA     0
## distance_maintained      0.92      0.94    0.88      0.94  15       NA     0
##                     med.r
## distancing           0.94
## distance_maintained  0.94
##
##  Item statistics
##                       n raw.r std.r r.cor r.drop mean   sd
## distancing          627  0.98  0.98  0.95   0.94  2.3 0.96
## distance_maintained 627  0.98  0.98  0.95   0.94  2.2 0.94
##
## Non missing response frequency for each item
##                        1    2    3 miss
## distancing          0.35 0.00 0.65    0
## distance_maintained 0.37 0.07 0.56    0
```

Internal Consistency - Isolation Strategies

```
isolate <- efa %>% dplyr::select(isolate_positive, isolate_symptoms, isolate_contact)
psych::alpha(isolate, title = "isolate", check.keys = TRUE)
```

```
##
## Reliability analysis  isolate
## Call: psych::alpha(x = isolate, title = "isolate", check.keys = TRUE)
##
##   raw_alpha std.alpha G6(smc) average_r S/N  ase mean   sd median_r
##       0.71      0.73    0.67      0.47 2.7 0.02  2.5 0.66     0.39
##
##  lower alpha upper     95% confidence boundaries
## 0.67 0.71 0.75
##
##  Reliability if an item is dropped:
##                  raw_alpha std.alpha G6(smc) average_r S/N alpha se var.r med.r
## isolate_positive      0.56      0.57    0.39      0.39 1.3    0.035    NA  0.39
## isolate_symptoms      0.54      0.55    0.38      0.38 1.2    0.035    NA  0.38
## isolate_contact       0.78      0.78    0.64      0.64 3.6    0.018    NA  0.64
##
##  Item statistics
##                    n raw.r std.r r.cor r.drop mean   sd
## isolate_positive 627  0.81  0.84  0.74   0.60  2.7 0.74
## isolate_symptoms 627  0.82  0.84  0.75   0.60  2.6 0.79
## isolate_contact  627  0.78  0.74  0.49   0.43  2.3 0.95
##
## Non missing response frequency for each item
##                     1    2    3 miss
## isolate_positive 0.16 0.01 0.82    0
## isolate_symptoms 0.19 0.01 0.80    0
## isolate_contact  0.35 0.01 0.64    0
```

Internal Consistency - Costs Incurred

```
financial <- efa %>% dplyr::select(paidtime_test_combined, paidtime_sick_combined, cost_test_com
bined)
psych::alpha(financial, title = "financial", check.keys = TRUE)
```

```
##
## Reliability analysis  financial
## Call: psych::alpha(x = financial, title = "financial", check.keys = TRUE)
##
##   raw_alpha std.alpha G6(smc) average_r S/N   ase mean   sd median_r
##        0.6       0.6    0.51      0.33 1.5 0.027  2.3 0.62     0.37
##
##  lower alpha upper     95% confidence boundaries
## 0.54 0.6 0.65
##
##  Reliability if an item is dropped:
##                        raw_alpha std.alpha G6(smc) average_r  S/N alpha se
## paidtime_test_combined      0.42      0.42    0.27      0.27 0.73    0.046
## paidtime_sick_combined      0.53      0.54    0.37      0.37 1.17    0.037
## cost_test_combined          0.54      0.54    0.37      0.37 1.15    0.037
##                        var.r med.r
## paidtime_test_combined    NA  0.27
## paidtime_sick_combined    NA  0.37
## cost_test_combined        NA  0.37
##
##  Item statistics
##                          n raw.r std.r r.cor r.drop mean   sd
## paidtime_test_combined 627  0.79  0.78   0.6   0.46  2.1 0.88
## paidtime_sick_combined 627  0.76  0.73   0.5   0.39  2.2 0.89
## cost_test_combined     627  0.69  0.73   0.5   0.38  2.6 0.72
##
## Non missing response frequency for each item
##                           1    2    3 miss
## paidtime_test_combined 0.32 0.21 0.47    0
## paidtime_sick_combined 0.33 0.19 0.48    0
## cost_test_combined     0.13 0.17 0.70    0
```

Internal Consistency - Number of Contacts

```
movement <- efa %>% dplyr::select(movement, bubbles, restrictedentry, risk)
psych::alpha(movement, title = "movement", check.keys = TRUE)
```

```
##
## Reliability analysis  movement
## Call: psych::alpha(x = movement, title = "movement", check.keys = TRUE)
##
##   raw_alpha std.alpha G6(smc) average_r S/N   ase mean   sd median_r
##        0.6       0.6    0.54      0.27 1.5 0.026  1.6 0.61     0.27
##
##  lower alpha upper     95% confidence boundaries
## 0.55 0.6 0.65
##
##  Reliability if an item is dropped:
##                 raw_alpha std.alpha G6(smc) average_r  S/N alpha se  var.r
## movement             0.44      0.45    0.36      0.22 0.82    0.038 0.0012
## bubbles              0.51      0.51    0.42      0.26 1.05    0.033 0.0044
## restrictedentry      0.58      0.58    0.48      0.31 1.36    0.028 0.0058
## risk                 0.57      0.57    0.48      0.31 1.32    0.030 0.0088
##                 med.r
## movement         0.21
## bubbles          0.29
## restrictedentry  0.29
## risk             0.31
##
##  Item statistics
##                   n raw.r std.r r.cor r.drop mean   sd
## movement        627  0.75  0.74  0.62   0.48  1.7 0.94
## bubbles         627  0.69  0.69  0.53   0.40  1.6 0.91
## restrictedentry 627  0.66  0.63  0.41   0.32  1.9 1.00
## risk            627  0.59  0.64  0.42   0.33  1.3 0.75
##
## Non missing response frequency for each item
##                    1    3 miss
## movement        0.67 0.33    0
## bubbles         0.70 0.30    0
## restrictedentry 0.54 0.46    0
## risk            0.83 0.17    0
```

Internal Consistency - Masking

```
masking <- efa %>% dplyr::select(masking1,mask_maintained)
psych::alpha(masking, title = "masking", check.keys = TRUE)
```

```
##
## Reliability analysis  masking
## Call: psych::alpha(x = masking, title = "masking", check.keys = TRUE)
##
##   raw_alpha std.alpha G6(smc) average_r S/N    ase mean   sd median_r
##        0.9       0.9    0.82      0.82 9.1 0.0079  2.5 0.81     0.82
##
##  lower alpha upper     95% confidence boundaries
## 0.89 0.9 0.92
##
##  Reliability if an item is dropped:
##                 raw_alpha std.alpha G6(smc) average_r S/N alpha se var.r med.r
## masking1             0.83      0.82    0.67      0.82 4.6       NA     0  0.82
## mask_maintained      0.81      0.82    0.67      0.82 4.6       NA     0  0.82
##
##  Item statistics
##                   n raw.r std.r r.cor r.drop mean   sd
## masking1        627  0.95  0.95  0.86   0.82  2.5 0.86
## mask_maintained 627  0.95  0.95  0.86   0.82  2.5 0.85
##
## Non missing response frequency for each item
##                    1    2    3 miss
## masking1        0.24 0.00 0.76    0
## mask_maintained 0.23 0.04 0.72    0
```

Criterion Validity

Concurrent Validity

Testing the hypothesis that overall protective scores/40 are significantly associated with the 'gold standard' question: Do you feel protected from COVID-19 at work? Group 1 = No or Unsure, Group 2 = Yes

Heterogeneity of variances verified- Bartlett test (p=0.55)

Normality verified -Shapiro-Wilks statistic (p <0.001)

Two-sample t-test shows that protective scores are significantly associated with the gold standard question 'Do you feel protected from COVID-19 at work?' (p < 0.001)

```
var.test(total ~ protected, data = all_protected)
```

```
##
##  F test to compare two variances
##
## data:  total by protected
## F = 1.0737, num df = 231, denom df = 350, p-value = 0.5473
## alternative hypothesis: true ratio of variances is not equal to 1
## 95 percent confidence interval:
##  0.8508462 1.3626183
## sample estimates:
## ratio of variances
##           1.073652
```

```
shapiro.test(all_protected$total)
```

```
##
##  Shapiro-Wilk normality test
##
## data:  all_protected$total
## W = 0.98954, p-value = 0.0003627
```

```
t.test(total ~ protected, data = all_protected)
```

```
##
##  Welch Two Sample t-test
##
## data:  total by protected
## t = -3.9007, df = 482.32, p-value = 0.0001095
## alternative hypothesis: true difference in means is not equal to 0
## 95 percent confidence interval:
##  -1.988956 -0.656417
## sample estimates:
## mean in group 1 mean in group 2
##        19.74569        21.06838
```

Construct Validity

Known Groups Technique

Total protective scores vary significantly between Canada and Ireland, the two largest participant groups (p = 0.04)

```
all_countries <- subset(all, all$country == "Canada" |all$country == "Ireland" )
table(all_countries$country)
```

```
##
##  Canada Ireland
##     243     210
```

```
t.test(total ~ country, data = all_countries)
```

```
##
##  Welch Two Sample t-test
##
## data:  total by country
## t = 1.9674, df = 423.68, p-value = 0.04979
## alternative hypothesis: true difference in means is not equal to 0
## 95 percent confidence interval:
##  0.000707231 1.542620223
## sample estimates:
##  mean in group Canada mean in group Ireland
##              21.02881              20.25714
```

Whether or not a worker feels protected from COVID-19 at work varies significantly by country (p=0.03)

(Countries with greater than 15 respondents included)

```
all_countries <- subset(all, all$country == "Argentina" |  all$country == "Canada" | all$country
== "China" |all$country == "Ireland" | all$country == "Nigeria" | all$country == "Poland" | all
$country == "United Kingdom of Great Britain and Northern Ireland")

table(all_countries$country, all_countries$protected)
```

```
##
##                                                          1   2
##   Argentina                                              5  16
##   Canada                                               106 134
##   China                                                  5  17
##   Ireland                                               72 114
##   Nigeria                                                6  11
##   Poland                                                 6  10
##   United Kingdom of Great Britain and Northern Ireland  27  17
```

```
chisq.test(all_countries$country, all_countries$protected)
```

```
##
##  Pearson's Chi-squared test
##
## data:  all_countries$country and all_countries$protected
## X-squared = 14.717, df = 6, p-value = 0.02257
```

Whether or not a worker feels protected from COVID-19 at work varies significantly by sector (p < 0.001)

(Sectors with greater than 20 respondents included)

```
all_sectors <- subset(all, all$industry == "Administration" | all$industry == "Construction" |al
l$industry == "Educational services" | all$industry == "Health care or social assistance" | all
$industry == "Professional,scientific or technical services" | all$industry == "Retail trade" |
 all$industry == "Manufacturing and food processing" | all$industry == "Public administration an
d defence" | all$industry == "Transportation or warehousing")

table(all_sectors$industry, all_sectors$protected)
```

```
##
##                                       1   2
##   Administration                      9  10
##   Construction                        5  10
##   Educational services               51  42
##   Health care or social assistance   87 148
##   Manufacturing and food processing   1  13
##   Public administration and defence  10  14
##   Retail trade                       18   7
##   Transportation or warehousing       9   7
```

```
chisq.test(all_sectors$industry, all_sectors$protected)
```

```
##
##  Pearson's Chi-squared test
##
## data:  all_sectors$industry and all_sectors$protected
## X-squared = 26.537, df = 7, p-value = 0.0004037
```

The health care and social assistance sectors have more protective measures in place than other occupational sectors (p < 0.001)

```
all$healthcare_not <- 0
all$healthcare_not[all$industry == "Health care or social assistance"] <- 1

var.test(total ~ healthcare_not, data = all)
```

```
##
##  F test to compare two variances
##
## data:  total by healthcare_not
## F = 0.90244, num df = 371, denom df = 254, p-value = 0.3679
## alternative hypothesis: true ratio of variances is not equal to 1
## 95 percent confidence interval:
##  0.7180225 1.1287293
## sample estimates:
## ratio of variances
##          0.9024378
```

```
shapiro.test(all$total)
```

```
##
##  Shapiro-Wilk normality test
##
## data:  all$total
## W = 0.9906, p-value = 0.0004994
```

```
t.test(total ~ healthcare_not, data = all)
```

```
##
##  Welch Two Sample t-test
##
## data:  total by healthcare_not
## t = -5.6044, df = 527.3, p-value = 3.373e-08
## alternative hypothesis: true difference in means is not equal to 0
## 95 percent confidence interval:
##  -2.511200 -1.207649
## sample estimates:
## mean in group 0 mean in group 1
##        19.45430        21.31373
```

Discriminant validity

No linear correlation between (1) total protective score and (2) improvement score (r=-0.05)

```
summary(all$score_improve)
```

```
##    Min. 1st Qu.  Median    Mean 3rd Qu.    Max.
##   0.000   0.000   2.000   2.096   3.000  10.000
```

```
corr.test(all$total,all$score_improve)
```

```
## Call:corr.test(x = all$total, y = all$score_improve)
## Correlation matrix
## [1] -0.05
## Sample Size
## [1] 627
## These are the unadjusted probability values.
##   The probability values  adjusted for multiple tests are in the p.adj object.
## [1] 0.2
##
##  To see confidence intervals of the correlations, print with the short=FALSE option
```

Significant negative linear correlation between (1) the number of measures workers would like to see improved and (2) feeling protected from COVID at work (r=-0.38)

```
corr.test(all$score_improve,all$protected)
```

```
## Call:corr.test(x = all$score_improve, y = all$protected)
## Correlation matrix
## [1] -0.38
## Sample Size
## [1] 583
## These are the unadjusted probability values.
##   The probability values  adjusted for multiple tests are in the p.adj object.
## [1] 0
##
##  To see confidence intervals of the correlations, print with the short=FALSE option
```

Significant correlation between (1) the total scores quartile and (2)

```
all$totalquart <- quantcut(all$total, q=4)
table(all$totalquart, all$protected)
```

```
##
##             1   2
##   [9,17]   71  57
##   (17,20]  69 103
##   (20,23]  49  99
##   (23,36]  43  92
```

```
chisq.test(all$totalquart, all$protected)
```

```
##
##  Pearson's Chi-squared test
##
## data:  all$totalquart and all$protected
## X-squared = 19.45, df = 3, p-value = 0.0002207
```

```
all$protected <- as.factor(all$protected)
logit <- glm(protected ~ totalquart, all, family = binomial)
logistic.display(logit, alpha=.05)
```

```
##
## Logistic regression predicting protected : 2 vs 1
##
##                          OR(95%CI)         P(Wald's test) P(LR-test)
## totalquart: ref.=[9,17]                                   < 0.001
##    (17,20]               1.86 (1.17,2.95)  0.009
##    (20,23]               2.52 (1.54,4.1)   < 0.001
##    (23,36]               2.67 (1.61,4.41)  < 0.001
##
## Log-likelihood = -382.2416
## No. of observations = 583
## AIC value = 772.4832
```

```
all$protected2 <- NA
all$protected2[all$protected == 2] <- 0
all$protected2[all$protected == 1] <- 1
all$protected2 <- as.factor(all$protected2)


all$totalquart = relevel(all$totalquart, ref=4)
logit2 <- glm(protected ~ totalquart, all, family = binomial)
logistic.display(logit2, alpha=.05)
```

```
##
## Logistic regression predicting protected : 2 vs 1
##
##                            OR(95%CI)         P(Wald's test) P(LR-test)
## totalquart: ref.=(23,36]                                    < 0.001
##    [9,17]                  0.38 (0.23,0.62)  < 0.001
##    (17,20]                 0.7 (0.43,1.12)   0.136
##    (20,23]                 0.94 (0.57,1.55)  0.822
##
## Log-likelihood = -382.2416
## No. of observations = 583
## AIC value = 772.4832
```

```
logit3 <- glm(protected ~ gender, all, family = binomial)
logistic.display(logit3, alpha=.05)
```

```
##
## Logistic regression predicting protected : 2 vs 1
##
##                                OR(95%CI)          P(Wald's test) P(LR-test)
## gender: ref.=Female                                              < 0.001
##    Male                        1.99 (1.35,2.94)   < 0.001
##    Non-binary / third gender 608349.25 (0,Inf)  0.98
##    Prefer not to say           0.13 (0.02,1.09)   0.06
##
## Log-likelihood = -380.4509
## No. of observations = 581
## AIC value = 768.9019
```

```
logit4 <- glm(protected ~ education, all, family = binomial)
logistic.display(logit4, alpha=.05)
```

```
##
## Logistic regression predicting protected : 2 vs 1
##
##                                             OR(95%CI)         P(Wald's test)
## education: ref.=College degree or higher
##    Prefer not to say                        0.48 (0.13,1.71)  0.257
##    Secondary school diploma                 0.84 (0.49,1.44)  0.534
##    Some secondary school education          1.64 (0.66,4.05)  0.288
##    Vocational training                      5.44 (2.1,14.08)  < 0.001
##
##                                             P(LR-test)
## education: ref.=College degree or higher < 0.001
##    Prefer not to say
##    Secondary school diploma
##    Some secondary school education
##    Vocational training
##
## Log-likelihood = -381.3728
## No. of observations = 583
## AIC value = 772.7456
```
