# Supplementary material for: Development and validation of a multi-lingual online questionnaire for surveying the COVID-19 prevention and control measures used in global workplaces
Source: BMC Public Health. 2022 Jan 12;22:74. doi: 10.1186/s12889-022-12500-w (PMC8753024; doi:10.1186/s12889-022-12500-w)
Supplement: Supplementary file 5 — Additional file 5. [file 12889_2022_12500_MOESM5_ESM.docx]

COVID Workplace Safety Survey

Start of Block: Default Question Block

Intro  (Español/Français/हिंदी/简体中文/Polski)
  How are you being protected from COVID-19 at work?    The survey will take about 5 minutes to complete. Your answers will help better protect the global workforce from COVID and future infectious disease outbreaks. Find out more info here: [Information Sheet](http://healthandagriscience.fra1.qualtrics.com/jfe/form/SV_9XZ98WvtyFMruHs)
 
By moving forward, you confirm that you agree with the following statements: 
 
I understand that my involvement in this study is voluntary and anonymous, and that my data will be stored securely. 


I am aged 18 or over.


I agree to take part in this University College Dublin survey.

| Page Break |  |
| --- | --- |

Q1 What is your age?

- Under 15 (1)
- 16 to 24 (2)
- 25 to 34 (3)
- 35 to 44 (4)
- 45 to 54 (5)
- 55 to 64 (6)
- 65 or over (7)

| Page Break |  |
| --- | --- |

Q2 What is your gender?

- Male (1)
- Female (2)
- Non-binary / third gender (3)
- Prefer not to say (4)

End of Block: Default Question Block

Start of Block: Country

| 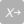 |
| --- |

Q3 In which country do you currently reside?

▼ Afghanistan (1) ... Zimbabwe (1357)

End of Block: Country

Start of Block: Education

Q4 What is your highest level of education?

- No formal education (1)
- Some primary school education (7)
- Some secondary school education (8)
- Secondary school diploma (2)
- College degree or higher (3)
- Vocational training (4)
- Prefer not to say (6)

End of Block: Education

Start of Block: Demographics Employment

Q5 Which category does your current work fall under?

- Formal working arrangement (1)
- Informal work (2)
- Self-employed (5)
- I am not currently working (4)
- Unsure (3)

Skip To: Q5a If Which category does your current work fall under? = I am not currently working

Display This Question:

If Which category does your current work fall under? = I am not currently working

Q5a If you're currently working full-time from home or looking for work, we apologize because the survey will now end abruptly. Because we're trying to see how workers are protected in the physical workplace, many of the questions won't be relevant to your current circumstances. We're so grateful for your participation all the same.


Before you go, we do have one last question. Are you vaccinated for COVID-19?

- Yes, fully (1)
- Yes, partially (2)
- No, I do not want to be vaccinated for COVID-19 (3)
- No, I have not yet had access to COVID-19 (4)

Skip To: End of Survey If If you're currently working full-time from home or looking for work, we apologize because the sur... = Yes, fully

Skip To: End of Survey If If you're currently working full-time from home or looking for work, we apologize because the sur... = Yes, partially

Skip To: End of Survey If If you're currently working full-time from home or looking for work, we apologize because the sur... = No, I do not want to be vaccinated for COVID-19

Skip To: End of Survey If If you're currently working full-time from home or looking for work, we apologize because the sur... = No, I have not yet had access to COVID-19

| Page Break |  |
| --- | --- |

Q6 Are you currently in a management role?

- Yes, Occupational Safety and Health management (1)
- Yes, other management (5)
- No (2)

| Page Break |  |
| --- | --- |

Display This Question:

If Which category does your current work fall under? = Formal working arrangement

| 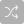 |
| --- |

Q7 Where are you employed?

- Private-for-profit company, business or individual, for wages, salary or commissions (1)
- Private-not-for-profit, tax-exempt, or charitable organization (2)
- Public institution employee/Civil servant (3)

| 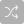 |
| --- |

Q8 Which of the following industries most closely matches the one in which you are employed?

- Forestry, fishing, hunting or agriculture support (1)
- Real estate or rental and leasing (2)
- Mining and quarrying (3)
- Professional, scientific or technical services (4)
- Electricity, gas, steam and air conditioning supply (5)
- Administration (6)
- Cleaning activities (21)
- Construction (7)
- Water supply; sewerage, waste management or remediation services (8)
- Manufacturing and food processing (9)
- Educational services (10)
- Wholesale trade (11)
- Health care or social assistance (12)
- Retail trade (13)
- Arts, entertainment or recreation (14)
- Transportation or warehousing (15)
- Accommodation or food services (16)
- Information and communication (17)
- Public administration and defence (18)
- Finance or insurance (19)
- Other services (20) ________________________________________________

Display This Question:

If Which of the following industries most closely matches the one in which you are employed? = Health care or social assistance

Q9 Which of the following settings most closely matches the one in which you are employed?

- Hospital (1)
- Long term care facility (2)
- Other health care/social care setting (3) ________________________________________________

Display This Question:

If Are you currently in a management role? = No

Q10 Which type of establishment do you currently work for?

- Small (less than 50 employees) (1)
- Mid-size (between 50-250 employees) (2)
- Large (more than 250 employees) (3)
- Unsure (7)

Display This Question:

If Are you currently in a management role? = Yes, Occupational Safety and Health management

Or Are you currently in a management role? = Yes, other management

Q11 Which type of establishment do you currently manage?

- Small (less than 50 employees) (1)
- Mid-size (between 50-250 employees) (2)
- Large (more than 250 employees) (3)
- Unsure (7)

Q12 Are you currently working from home?

- Yes, full time (1)
- Yes, partially (2)
- No (3)

Skip To: Q12a If Are you currently working from home? = Yes, full time

Display This Question:

If Are you currently working from home? = Yes, full time

Q12a If you're currently working full-time from home or looking for work, we apologize because the survey will now end abruptly. Because we're trying to see how workers are protected in the physical workplace, many of the questions won't be relevant to your current circumstances. We're so grateful for your participation all the same. 


 Before you go, we do have one last question. Are you vaccinated for COVID-19?

- Yes, fully (1)
- Yes, partially (2)
- No, I do not want to be vaccinated for COVID-19 (3)
- No, I have not yet had access to a COVID-19 vaccine (4)

Skip To: End of Survey If If you're currently working full-time from home or looking for work, we apologize because the sur... = Yes, fully

Skip To: End of Survey If If you're currently working full-time from home or looking for work, we apologize because the sur... = Yes, partially

Skip To: End of Survey If If you're currently working full-time from home or looking for work, we apologize because the sur... = No, I do not want to be vaccinated for COVID-19

Skip To: End of Survey If If you're currently working full-time from home or looking for work, we apologize because the sur... = No, I have not yet had access to a COVID-19 vaccine

End of Block: Demographics Employment

Start of Block: Basic IPC Measures

Q13 Which prevention and control measures, that you know of, are currently in place in your workplace in response to COVID-19? (Select all that apply)

- Increased hand hygiene (1)
- Frequent disinfection of touched objects and surfaces (2)
- Social distancing (> 1 metre) between employees (4)
- Masking of all employees (5)
- Limiting employee movement between facilities (7)
- Worker bubbles (i.e., employees work with same, small group of colleagues at all times) (8)
- Facility entry restrictions (i.e., only essential personnel may enter) (9)
- High-risk employees (i.e., > 60 yrs old, underlying health conditions) work from home (10)

Display This Question:

If Which prevention and control measures, that you know of, are currently in place in your workplace... = Social distancing (> 1 metre) between employees

Or Which prevention and control measures, that you know of, are currently in place in your workplace... = Masking of all employees

Or Which prevention and control measures, that you know of, are currently in place in your workplace... =

Q14 How often would you say the following statements are true?

Display This Choice:

If Which prevention and control measures, that you know of, are currently in place in your workplace... = Social distancing (> 1 metre) between employees

Display This Choice:

If Which prevention and control measures, that you know of, are currently in place in your workplace... = Masking of all employees

Or Which prevention and control measures, that you know of, are currently in place in your workplace... =

|  | Always (1) | Sometimes (2) | Rarely (3) | Never (4) |
| --- | --- | --- | --- | --- |
| Display This Choice:  If Which prevention and control measures, that you know of, are currently in place in your workplace... = Social distancing (> 1 metre) between employees  I am able to maintain a social distance of at least 1 metre at work (1) |  |  |  |  |
| Display This Choice:  If Which prevention and control measures, that you know of, are currently in place in your workplace... = Masking of all employees  Or Which prevention and control measures, that you know of, are currently in place in your workplace... =  Those I come into contact with at work wear their mask correctly (2) |  |  |  |  |

End of Block: Basic IPC Measures

Start of Block: Environmental Adjustments

Q15 Which of the following environmental adjustments, that you know of, are currently in place in your workplace in response to COVID-19? (select all that apply)

- Temperature adjustments (1)
- Ventilation adjustments (2)
- Environmental monitoring (i.e., testing touched objects and surfaces for SARS-CoV-2) (3)
- Air quality monitoring (7)
- Unsure (5)
- None (6)

End of Block: Environmental Adjustments

Start of Block: Surveillance

Q16 Which of these measures have been used in your workplace in the last three months?

- Symptomatic employees (1)
- Employees who come into contact with someone with COVID (2)
- All employees at once (3)
- Random groups of employees (5)
- They come into contact with someone with COVID-19 (6)
- They have COVID-19 symptoms (8)
- They test positive for COVID-19 (7)
- Employees get their temperature checked (9)
- Employees with COVID-19 symptoms are reported to management (4)
- Unsure (11)
- None of the above (10)

| Page Break |  |
| --- | --- |

Display This Question:

If Are you currently in a management role? = Yes, Occupational Safety and Health management

Or Are you currently in a management role? = Yes, other management

Q17a Where would employees seek a COVID-19 test? (Select all that apply)

- Testing provided in the workplace by employer (1)
- Testing provided outside of workplace in private facility (2)
- Testing provided outside of workplace in public facility (3)
- Unsure (5)

Display This Question:

If Are you currently in a management role? = No

Q17b Where would you seek a COVID-19 test? (Select all that apply)

- Testing provided in the workplace by employer (1)
- Testing provided outside of workplace in private facility (2)
- Testing provided outside of workplace in public facility (3)
- Unsure (5)

| Page Break |  |
| --- | --- |

Display This Question:

If Are you currently in a management role? = Yes, Occupational Safety and Health management

Or Are you currently in a management role? = Yes, other management

Q18a Who covers the cost of employee COVID-19 tests?

- Employer (1)
- Government (2)
- Employee (3)
- Other (4) ________________________________________________
- Unsure (5)

Display This Question:

If Are you currently in a management role? = No

Q18b Who covers the cost of a work-related COVID-19 test?

- Employer (1)
- Government (2)
- Employee (3)
- Other (4) ________________________________________________
- Unsure (5)

Display This Question:

If Are you currently in a management role? = Yes, Occupational Safety and Health management

Or Are you currently in a management role? = Yes, other management

Q19a Do employees receive paid time off to be tested for COVID?

- Yes (1)
- No (2)
- Unsure (3)

Display This Question:

If Are you currently in a management role? = No

Q19b Do you receive paid time off to be tested for COVID?

- Yes (1)
- No (2)
- Unsure (3)

Display This Question:

If Are you currently in a management role? = Yes, Occupational Safety and Health management

Or Are you currently in a management role? = Yes, other management

Q20a Do employees receive paid sick leave if they have COVID?

- Yes (1)
- No (2)
- Unsure (3)

Display This Question:

If Are you currently in a management role? = No

Q20b Do you receive paid time off if you have COVID?

- Yes (1)
- No (2)
- Unsure (3)

| Page Break |  |
| --- | --- |

Display This Question:

If Which of these measures have been used in your workplace in the last three months? = All employees at once

Or Which of these measures have been used in your workplace in the last three months? = Random groups of employees

Or Which of these measures have been used in your workplace in the last three months? = Employees get their temperature checked

Q21 How frequently would you say the following screening measures are used in your workplace?

Display This Choice:

If Which of these measures have been used in your workplace in the last three months? = All employees at once

Display This Choice:

If Which of these measures have been used in your workplace in the last three months? = Random groups of employees

Display This Choice:

If Which of these measures have been used in your workplace in the last three months? = Employees get their temperature checked

|  | Once or a few times (1) | Regularly (monthly, weekly, etc.) (2) | Following an outbreak (3) |
| --- | --- | --- | --- |
| Display This Choice:  If Which of these measures have been used in your workplace in the last three months? = All employees at once  Testing of all employees, even those without symptoms (1) |  |  |  |
| Display This Choice:  If Which of these measures have been used in your workplace in the last three months? = Random groups of employees  Testing of randomly selected employees, even those without symptoms (2) |  |  |  |
| Display This Choice:  If Which of these measures have been used in your workplace in the last three months? = Employees get their temperature checked  Employee temperature checks (3) |  |  |  |

End of Block: Surveillance

Start of Block: Contact tracing

Q22a Does your workplace use a contact tracing program?

- Yes (1)
- No (2)
- Unsure (3)

Skip To: End of Block If Does your workplace use a contact tracing program? = No

Skip To: End of Block If Does your workplace use a contact tracing program? = Unsure

Q22b If yes, who sponsors the contact tracing program(s)? (Select all that apply)

- Company/employers (1)
- Government (2)
- Unsure (3)

Skip To: Q29 If If yes, who sponsors the contact tracing program(s)? (Select all that apply) = Unsure

| Page Break |  |
| --- | --- |

Display This Question:

If If yes, who sponsors the contact tracing program(s)? (Select all that apply) = Company/employers

Q23 Have you ever been contacted by your company's contact tracing network?

- Yes (1)
- No (2)
- Unsure (3)

Skip To: Q26 If Have you ever been contacted by your company's contact tracing network? = Unsure

Skip To: Q26 If Have you ever been contacted by your company's contact tracing network? = No

Display This Question:

If Have you ever been contacted by your company's contact tracing network? = Yes

Q24 If yes, how were you contacted? (Select all that apply)

- Phone call (1)
- Email (2)
- Contact tracing application (3)
- Other (4) ________________________________________________

Display This Question:

If Have you ever been contacted by your company's contact tracing network? = Yes

Q25 How much time would you estimate passed between coming into contact with a COVID-19 case and being notified by your company's contact tracing system?

- Less than 24 hours (1)
- Between 24 and 47 hours (2)
- Between 48 and 72 hours (3)
- More than 72 hours (4)
- Unsure (5)

| Page Break |  |
| --- | --- |

Display This Question:

If If yes, who sponsors the contact tracing program(s)? (Select all that apply) = Government

Q26 Have you ever been contacted by a government contact tracing network?

- Yes (1)
- No (2)
- Unsure (3)

Skip To: Q29 If Have you ever been contacted by a government contact tracing network? = Unsure

Skip To: Q29 If Have you ever been contacted by a government contact tracing network? = No

Display This Question:

If Have you ever been contacted by a government contact tracing network? = Yes

Q27 If yes, how were you contacted? (Select all that apply)

- Phone call (1)
- Email (2)
- Contact tracing application (3)
- Other (4) ________________________________________________

Display This Question:

If Have you ever been contacted by a government contact tracing network? = Yes

Q28 How much time would you estimate passed between coming into contact with a COVID-19 case and being notified by the government's contact tracing system?

- Less than 24 hours (1)
- Between 24 and 47 hours (2)
- Between 48 and 72 hours (3)
- More than 72 hours (4)
- Unsure (5)

| Page Break |  |
| --- | --- |

Q29 Have you come into contact with a known COVID-19 case in the workplace and/or been diagnosed positive for COVID-19 without ever being contacted by a contact tracing system?

- Yes (1)
- No (2)
- Unsure (3)

End of Block: Contact tracing

Start of Block: PPE

Display This Question:

If Which of the following industries most closely matches the one in which you are employed? = Health care or social assistance

Q30 Which Personal Protective Equipment (PPE), that you know of, is used in your workplace in response to COVID-19?

- Surgical masks (1)
- Cloth masks (2)
- Respirators used for aerosol generating procedures (3)
- Gowns (4)
- Gloves (6)

Display This Question:

If Which of the following industries most closely matches the one in which you are employed? = Health care or social assistance

Q31 Does your workplace experience shortages of PPE?

- Always (1)
- Sometimes (2)
- Rarely (3)
- Never (4)

End of Block: PPE

Start of Block: Education

Q32 Which of the following education initiatives, that you know of, have been implemented in your workplace in response to COVID-19?

- COVID-19 signage in one language (1)
- COVID-19 signage in multiple languages (2)
- Training on proper use of PPE (3)
- Training on how to safely interact with colleagues in work (4)
- Training on how to safely socialize outside of work (6)
- Other (7) ________________________________________________

End of Block: Education

Start of Block: Follow up

Q45 Do you feel protected from COVID-19 at work?

- Yes (1)
- No (2)
- Unsure (4)

Q33 Which, if any, of the following do you feel need to be improved in your workplace in order to protect workers from COVID-19?

- Implementation of basic preventive measures by management (hand hygiene, masking, social distancing) (1)
- Access to preventive resources (shortages of masks, handwashing, physical barriers, etc.) (11)
- Access to funding for preventive measures (12)
- Adherence to basic preventive measures by employees (4)
- Worker education and training (2)
- Contact tracing (3)
- COVID-19 testing (5)
- Access to COVID-19 vaccines (7)
- Environmental Adjustments (temperature, ventilation) (6)
- Other (10) ________________________________________________
- None of the above (13)

Q34 Have you been vaccinated for COVID-19?

- Yes, fully (1)
- Yes, partially (2)
- No, I prefer not to be vaccinated for COVID-19 (3)
- No, I have not yet had access to a COVID-19 vaccine (4)

Q35 Which, if any, of the following measures should be removed from your workplace once most people have been vaccinated?

- Masking (1)
- Social distancing (11)
- Handwashing stations (12)
- Worker education and training on COVID-19 (2)
- Contact tracing (3)
- COVID-19 testing (5)
- Temperature and ventilation adjustments (6)
- Other (10) ________________________________________________
- None of the above (13)

End of Block: Follow up
