# Supplementary material for: Development and validation of a multi-lingual online questionnaire for surveying the COVID-19 prevention and control measures used in global workplaces
Source: BMC Public Health. 2022 Jan 12;22:74. doi: 10.1186/s12889-022-12500-w (PMC8753024; doi:10.1186/s12889-022-12500-w)
Supplement: Supplementary file 6 — Additional file 6. [file 12889_2022_12500_MOESM6_ESM.docx]

Information Sheet

Who is conducting this survey?

A research team from the School of Public Health, Physiotherapy, and Sports Science at University College Dublin, led by Dr. Carla Perrotta and Dr. Conor Buggy in affiliation with the Centre for Health and Safety at Work. 

 Who sponsors this research? 

This research project is funded by the Science Foundation Ireland COVID-19 Rapid Response Programme - Grant 20/COV/8539.

What’s the purpose of this survey?

Research shows that some workplaces are common settings for infectious disease outbreaks due to transmission between employees, their households, and communities. Because employees have a legal right to a safe workplace, employers must do their best to create safe work conditions.   From February to April 2021, our University College Dublin team and partners searched through ~14,000 research articles to identify which safety measures effectively prevent COVID-19 outbreaks in the workplace. Compiling evidence from international healthcare, nursing home, meatpacking, manufacturing, and office settings, we found that comprehensive measures incorporating swift and thorough contact tracing, effective Personal Protective Equipment (PPE), regular testing, and worker bubbles can prevent viral transmission. Masking alone, we learned, is not sufficient protection in workplaces at high risk of transmission.

Considering these findings, our research team now seeks to understand where effective occupational COVID-19 safety measures are/are not being implemented internationally using a multi-lingual online survey. Survey findings will provide insight into how to better protect workers, particularly in environments where infections remain uncontrolled. They will also benefit future occupational preparedness against viral disease outbreaks.

Over fifteen months into the pandemic, some countries have yet to issue occupational COVID-19 safety regulations. We hope that this survey will help to raise awareness towards individuals’ right to safe work conditions, and what these entail.

Find more information on our research project here: [Workplace COVID-19 Prevention and Control Measures: Rapid Review and Meta-analysis](http://osf.io/vn7x6/), [Research Protocol](https://osf.io/87ksm)

What will my participation involve?

The survey will take about 5 minutes to complete. At no point will we ask you any personal, identifying information. The questions start with some general information about you and where you work and move on to questions about COVID safety measures in your workplace.

Who can take part?

Anyone over the age of 18 can take part, though we ask that you are currently, actively working. If you are working full-time from home, you are still welcome to participate but we won’t ask you any questions about safety measures in your workplace.

What will happen to the information I give you?

All data will be collected and stored anonymously on QualtricsXM, an online survey platform.   Access to Qualtrics systems is restricted to specific individuals who have a need-to-know such information and who are bound by confidentiality obligations. Access is monitored and audited for compliance. More information on Qualtrics security and privacy standards is available here: **https://www.qualtrics.com/platform/security/**

For analysis purposes, your anonymous information will be downloaded to an encrypted, password protected University College Dublin PC with a specific IP address. This PC, and all anonymous information stored on it, can only be accessed by members of the research team.   Findings will be shared with researchers, occupational safety and health professionals, and policymakers. We won’t be able to identify or contact you but are more than happy to share survey results if you contact us at **workingwithcovid@ucd.ie**. You are also welcome to contact us at any time with questions regarding the use of your information and/or your rights in relation to this information.

How do I withdraw?

Because this survey is anonymous, it will not be possible to remove your answers after they have been submitted.

Ethics approval

This research project has been approved for exemption from full ethical review by UCD Human Research Ethics Committee – Sciences based on its status as a low-risk product. Reference: LSE-21-138 Perrotta.
